# Image-Based Volatile Organic Compound Identification Using the Cosine Similarity Method

**DOI:** 10.1021/acsomega.6c00450

**Published:** 2026-05-19

**Authors:** Jingqin Mao, Zhenxun Wu, Seán McLoone, Hamza Shakeel

**Affiliations:** † School of Electronics, Electrical Engineering and Computer Science, 1596 Queen’s University Belfast, Belfast BT7 1NN, U.K.; ‡ Department of Science, Technology, Engineering and Public Policy, 4919 University College London, Gower Street, London WC1E 6BT, U.K.

## Abstract

Accurate detection and identification of volatile organic compounds (VOCs) is critical for environmental monitoring, industrial safety, and human health. Currently, microsensor-based VOC detection techniques mainly rely on electrical or frequency-based readout signals, which cannot be used for the identification of chemicals. Herein, we present a visual and intuitive method for identifying VOCs. Our technique is based on the analysis of images generated by photoionization of gas samples using a helium-based dielectric plasma detector. The helium plasma color change upon sample injection is recorded by a standard phone camera. The specific plasma images corresponding to VOC injections are extracted from the recorded videos. VOCs (*n*-pentane (C5), benzene, acetone) from three different classes (alkanes, polar organic compounds, aromatics) and their mixtures (C5-benzene, C5-acetone, benzene-acetone) are tested at four different injection pressures (17, 20, 24, 27 Psi). All the obtained plasma images are divided into test and training data sets, and the cosine similarity method is then used to identify the test data set images. The image feature vectors for cosine similarity calculation are obtained by direct image segmentation. Moreover, the impact on identification accuracy, including different regions of the image is also studied, revealing a sensitivity to image regions. Results show that VOC identification accuracy is close to 100% when using the cosine similarity method based on direct image segmentation. This work demonstrates a new approach for VOC identification through plasma image analysis and has the potential to facilitate the development of low-cost VOC sensors.

## Graphical abstract

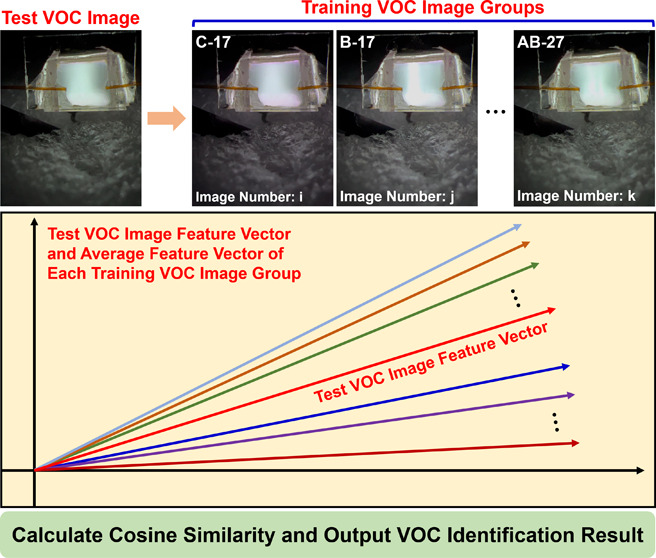

## 1 Introduction

Volatile organic compounds (VOCs) usually have relatively high vapor pressure at ambient temperature and widely exist in the environment.^1^ Both human activities and natural environment processes are the main sources of emissions of VOCs.^2^ Most common VOCs, such as benzene, acetone, and formaldehyde, are highly toxic to humans.^3^ In addition, VOCs can also have negative impact on the environment, such as causing atmospheric ozone layer depletion and harming the growth of crops.^4^ Therefore, it is very important to detect and identify VOCs. Gas chromatograph (GC) is a common and reliable analytical instrument for the detection and identification of VOCs.^5–7^ Moreover, gas chromatography–mass spectrometry (GC–MS) is also considered the gold standard for analysis of VOCs.^8–10^ However, traditional GC systems are usually expensive, bulky, and require high power.^11^ Furthermore, GC–MS systems are more expensive, larger, and more complex to operate than conventional GC systems, making them unsuitable for portable and on-site VOCs detection and identification.^12^ In addition to the GC/GC–MS techniques, various alternative analytical techniques have been investigated for detection of VOCs, including electrochemical sensors,^13,14^ metal oxide sensors,^15,16^ quartz crystal microbalance (QCM) sensors,^17–19^ surface acoustic wave (SAW) sensors,^20,21^ photoionization detectors (PIDs),^22,23^ electronic nose,^24,25^ optical sensors,^1,26^ and micro gas chromatography (μGC) systems.^27–29^ These techniques provide promising options for low-cost, on-site, and real-time detection of VOCs. A detailed comparison of working principles, advantages, and disadvantages of these techniques is summarized in Table S1.

Among these techniques, the μGC system is an excellent candidate for on-site and real-time detection of VOCs due to the advantages of being portable, low cost, and low power.^30–32^ A typical μGC configuration includes a carrier gas source, sampling device (preconcentrator), separation column, detector, pump and valve system, and software for control and data processing.^27,33^ Among these components, the detector is critical since it determines the quality and type of μGC system signal output. Some common gas detector technologies that have been used in μGC systems include photoionization detectors (PID),^22,34,35^ thermal conductivity detectors (TCD),^36–39^ flame ionization detectors (FID),^40–42^ electron capture detectors (ECD),^43,44^ capacitive detectors,^45–47^ chemiresistor detector arrays,^48–51^ surface acoustic wave (SAW) sensors,^52,53^ film bulk acoustic resonators (FBAR),^54,55^ optical ring resonators,^56,57^ and nanoelectromechanical resonators.^58,59^ Currently, these detectors mainly rely on electrical or frequency signals as outputs to detect VOCs. The detection process of VOCs using these technologies is invisible to the operator and requires the use of known calibration standards before analysis and identification of the unknown sample. In our previous work, we reported a colorimetric signal readout-based micro helium dielectric barrier discharge photoionization detector (μHDBD-PID) that utilizes the change in plasma color upon injection of VOCs into the plasma chamber as a readout signal for detection. The plasma color change was first recorded by a smartphone camera and then converted to image light intensity versus retention time plots (gas chromatograms) using three standard color space models (red, green, blue (RGB); hue, saturation, lightness (HSL); and hue, saturation, value (HSV)).^60^ However, this method also cannot identify VOCs without connecting a GC column or using other analytical techniques, such as spectrometry to measure the optical emission spectrum (OES). In our earlier work, we combined the μHDBD-PID with a mini spectrometer to detect and identify VOCs based on the OES of different classes of compounds.^61^ It is worth noting that plasma color changes are clearly visible during the ionization process and more importantly, different VOCs produce different plasma colors. Therefore, in our current work, we use this phenomenon as a direct method of identifying different classes of VOC.

Image classification based on computer vision has been widely used for various applications, including medical diagnosis,^62^ food safety,^63^ plant disease analysis,^64^ insect classification,^65^ remote environmental sensing,^66^ and vehicle classification.^67^ The classification methods used in computer vision are generally universal across application domains as they rely on the information extracted through image analysis. Therefore, it is feasible to use computer vision-based methods to conduct plasma image recognition, and several studies have already been demonstrated.^68–73^ In 2022, Falato et al. reported the classification of laboratory-obtained astrophysical plasma jets using a cosine similarity constrained convolutional neural network (CNN) method and plasma images.^74^ This work developed a simple vector based comparison algorithm using the cosine similarity method and achieved approximately 92% accuracy on binary and five-way plasma image classification. The cosine similarity method is used to compare the similarity between vectors by calculating the cosine value of the angle between two vectors. The closer the cosine value of the two vectors is to 1, the higher the similarity between them.^75^ Cosine similarity is widely used in text detection, such as document similarity detection, text classification and web page data mining.^76–80^

For image recognition, vectors can be extracted from images directly or created artificially based on different recognition requirements. Published research on VOC identification using cosine similarity is mainly based on MS and GC spectral data, with no prior research reported on VOC identification based on plasma images using PIDs.^81–84^ In this work, we present a method for identifying VOCs based on μHDBD-PID plasma images using the cosine similarity method. The whole process of VOCs entering the chamber and being ionized is recorded by a camera, and an image with a characteristic plasma color is then extracted. The plasma images are then converted to a vector by a computer algorithm to obtain the feature vectors. Afterward, we use the cosine similarity method to calculate the similarity between these feature vectors and output the results.

The image feature vectors in this study are constructed by using the RGB information extracted from plasma images. The image is segmented into a set number of square blocks, and the average RGB values of all pixels in each block are calculated. Each block generates a group of average RGB values, which are then combined to construct the feature vector. We also studied the effects of using different image regions and the number of blocks on the classification results. A higher number of blocks after performing image segmentation corresponds to a higher image resolution. The results demonstrate that the cosine similarity method achieves excellent classification accuracy and low computation time when using a small number of large-sized blocks (relatively low resolution). Computation time increases significantly as block size decreases (resolution improves with more segmented blocks) but classification accuracy does not improve. To the best of our knowledge, there is currently no work reported on VOC recognition based on plasma images generated by μPIDs. As such, this work demonstrates the feasibility of employing low cost RGB cameras and computer vision methods for identification of VOCs.

## 2 Experimental Section

### 2.1 Materials

N-pentane (C5), acetone (≥99.8%), and benzene were ordered from Sigma-Aldrich (U.K.) and used as received without any purification. The high purity compressed helium gas cylinder (99.999% purity, grade zero, N5.0) used to generate helium plasma was purchased from BOC Gases (U.K.). The Polydimethylsiloxane (PDMS) and PDMS hardener kit (SYLGARD 184) were bought from Dow Silicones (U.K.). The Glassomer glass casting solution (UV-L50) and its specific hardener were purchased from Glassomer GmbH (Germany). The transparent acrylic sheet (thickness: 3 mm) used to fabricate the ultraviolet (UV)-cured Glassomer plasma chamber under UV light in the clean room darkroom (isolate UV light) was bought from Rapid Electronics (U.K.) and cut by the technical support staff. The silver conductive epoxy adhesive (Loctite Hysol 9492 and 8331 adhesive) that is used to connect metal electrodes to the plasma chamber electrode surfaces and heat sink compound (helps promote heat dissipation) were purchased from RS Components Ltd. (U.K.). The high transparency and ultrathin circular glass slides (diameter: 30 mm, thickness: 0.17 mm) used as the helium plasma observation window and plasma chamber dielectric layer (bottom) were purchased from Thermo Scientific (U.K.). UV curable glue (Norland Optical Adhesive 81) which is used to bond the glass observation window to the plasma chamber was ordered from Edmund Optics (U.K.). The fused silica capillary tube (inner diameter: 0.15 mm) which is used to connect the plasma chamber inlet and the GC system was bought from Molex (U.K.).

### 2.2 Power Supply Design and Plasma Chamber Fabrication

The power supply used in this study is the same as our earlier published work.^60,61^ The plasma chamber fabrication process and μHDBD-PID used in this study were also same as reported in our previous work.^60^

### 2.3 VOCs Injection and Plasma Image Data Acquisition

Three VOCs (C5, benzene, and acetone) and their equal volume mixtures (C5-benzene, C5-acetone, and benzene-acetone) were tested in this study. All VOCs and VOC mixtures were manually injected in liquid form through the GC system (Agilent 7820A) injection port under split mode (1:200) and pushed into the helium plasma chamber through the fused silica capillary tube. The injection volume for each single VOC is 0.4 μL, and the injection volume for a VOC mixture is 0.8 μL. The high purity helium is used as both a carrier gas and helium plasma gas source. The GC injection port inlet temperature was set to 270 °C and the GC inlet pressure was set to four different values (17, 20, 24, and 27 Psi) to explore the plasma image differences corresponding to VOC injection under different pressures. An iPhone 13 Pro smartphone was used to record the entire process of plasma color change during VOC injection. The plasma videos are then processed using the image processing toolbox of MATLAB to extract frame by frame plasma images.^60^

### 2.4 Image Data Process

Due to the relatively small data size, we selected one plasma image during VOC injection from each category (24 images) to form the test data set for VOC classification. The remaining images are used as a training data set (95 images). The same operation is applied to helium plasma images. Four images constitute the test data set, and 24 images are used as the training data set. We cropped all the images into three image regions based on the plasma image content for RGB based image feature vector generation. Region 1 images (1080 × 1350 pixels) correspond to the image area after cropping the top and bottom black background areas from the original image. Region 2 images (360 × 324 pixels) focus on the glass plasma observation window area of the image. Region 3 images (540 × 400 pixels) are cropped from the bottom area of the original image where there is faint light reflection from the generated plasma.

### 2.5 VOC Classification Based on Images

The test and training images are placed in specific folders after preprocessing, where they are then used by the developed classification algorithm to output the classification results. The python code for the classification algorithm is included in the Supporting Information. The algorithm first calculates the average feature vector for each image category. Then it calculates the cosine similarity between the test image feature vectors and the average feature vectors of the 24 categories of training data set images. Finally, the algorithm outputs the classification results based on similarity.

## 3 Results and Discussion

### 3.1 Plasma Images during VOC Injection

The injection of VOCs causes changes to both plasma color and shape, and this change is usually very quick (∼1 s). The plasma color change will go from weak to strong, then from strong to weak until it changes back to the base helium plasma color within dozens of frames. The plasma color related to the peak plasma image light intensity is very stable for the same VOCs. A specific plasma image that corresponds to the maximum ionization of the VOCs will usually appear repeatedly near the peak light intensity. This image can be observed for one to three frames around the peak image light intensity under different tests when the same VOC is injected. Because the fully saturated plasma images for different VOCs have obvious differences, we selected the fully saturated plasma images as the primary data for this work. Additionally, the plasma images of the same VOCs at different pressures are also different. The reason for the difference is that the ionization ability of μHDBD-PID varies with pressure. In this study, C5, benzene, acetone, and their equal volume mixtures (C5-benzene, C5-acetone, and benzene-acetone) were tested at four inlet pressures (17 Psi, 20 Psi, 24 Psi, and 27 Psi), resulting in a total of 24 plasma image categories. The test was repeated at least 6 times for each category and the image acquisition method is the same as our previously published work.^60^ For some VOCs/VOC mixtures, the plasma image corresponding to detector saturation was not obtained in every test. Therefore, 119 images with stable plasma color were selected. Among them, 24 images were selected as the test data set, and the remaining 95 images were used as the training data set. As shown in Table 1, we labeled the plasma image categories using the initials of the VOC/VOC mixtures and the inlet pressures to facilitate classification.

**Table 1:** Allocated Image Identifiers Corresponding to Six VOCs and Mixtures Tested at Four Different Pressure Values

	C5	benzene	acetone	benzne + C5	acetone + C5	acetone + benzene
17 Psi	C-17	B-17	A-17	BC-17	AC-17	AB-17
20 Psi	C-20	B-20	A-20	BC-20	AC-20	AB-20
24 Psi	C-24	B-24	A-24	BC-24	AC-24	AB-24
27 Psi	C-27	B-27	A-27	BC-27	AC-27	AB-27

Figure 1 presents the original plasma images when different VOCs are injected into the plasma chamber. It can be easily seen that different VOCs produce different plasma colors and color distribution, and that slight changes in color are also evident for the same VOCs at different pressures. It is worth mentioning that the temperature of μHDBD-PID during operation can reach close to ∼103 °C,^60^ and the ambient temperature fluctuations of ±3 °C observed during testing had no effect on plasma operation. Additionally, the sample is injected using a GC system which maintains the injector temperature at 270 °C. The heated and vaporized sample also does not affect device performance. The results show that for VOCs like C5 (Figure 1—C-17, C-24, C-24, C-27), acetone (Figure 1—A-17, A-20, A-24, A-27), and acetone + C5 (Figure 1—AC-17, AC-20, AC-24, AC-27) only the plasma color changes. The color distribution of the plasma is not affected. In contrast, the injection of benzene (Figure 1—B-17, B-20, B-24, B-27), benzene + C5 (Figure 1—BC-17, BC-20, BC-24, BC-27), and acetone + benzene (Figure 1—AB-17, AB-20, AB-24, AB-27) result in changes in both the plasma color and its distribution. The change in plasma color distribution corresponding to benzene and its mixtures can be attributed to the relatively low limit of detection (LoD) of μHDBD-PID for benzene. The LoDs of μHDBD-PID for the three VOCs (C5, benzene, and acetone) are 316, 10, and 26 ng (20 Psi), respectively, when using the colorimetric signal readout method.^60^ With VOC mixtures, the LoD will fall between the LoDs of the two individually injected VOCs.^60^ The detector is easily saturated when a high amount of benzene (>100 ng) is injected, resulting in a change in the color distribution of plasma due to the limited ionization ability of μHDBD-PID. The detector has a relatively large linear range for C5 and acetone compared to benzene.^60^ Therefore, the plasma color distribution is reduced when they are mixed with benzene and injected, especially for C5 + benzene. The saturation of the detector has no effect on the characteristic plasma color of the image light intensity peak. The characteristic colors of the injected VOCs will still appear stably despite the saturation, such as the light green tint for benzene. In addition, the changes in plasma color distribution increases the differences between images, which may further facilitate the classification process.

**Figure 1:**
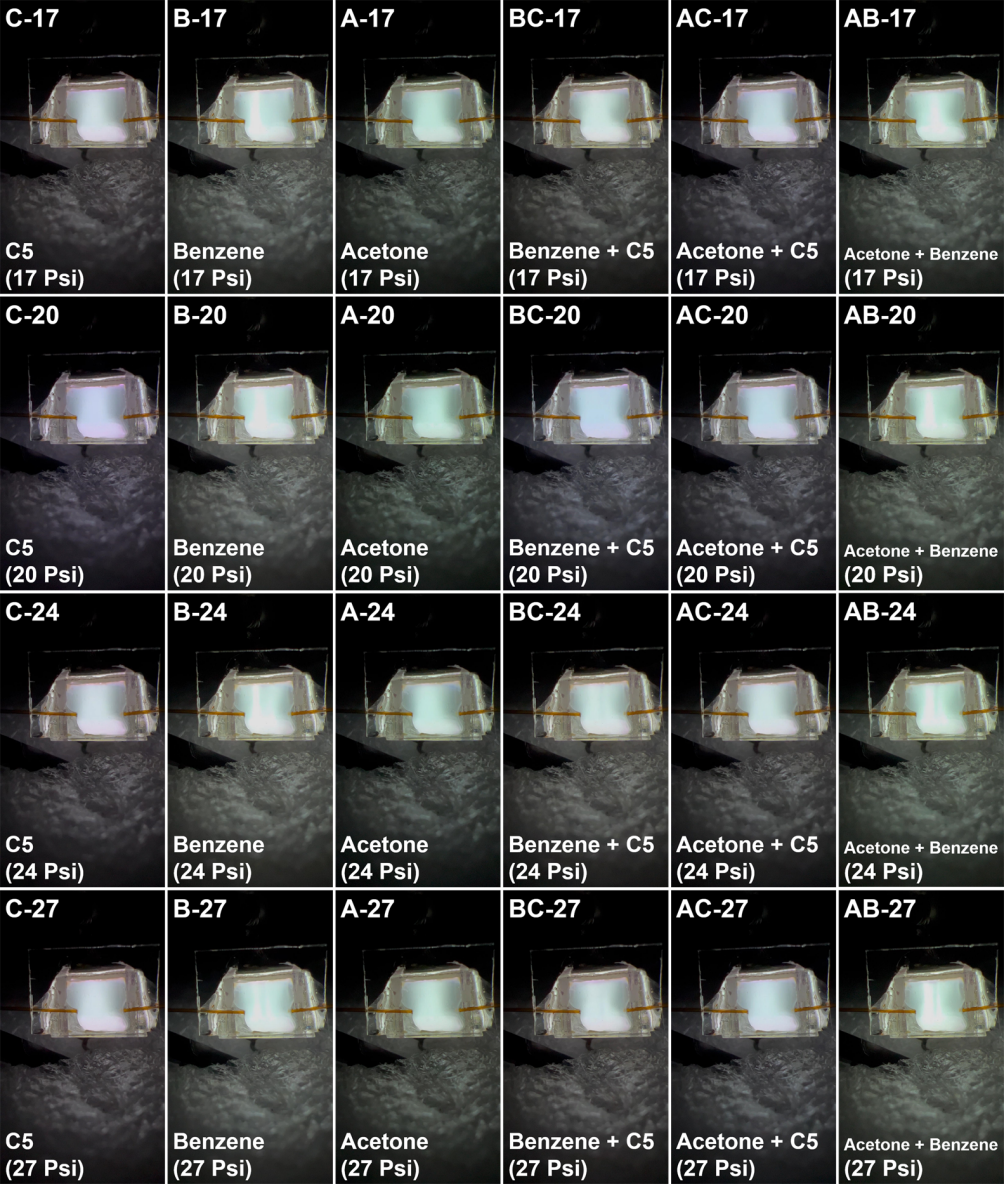
Plasma color changes corresponding to the injection of different VOCs (mixtures) into the plasma chamber at different inlet pressures. The four tested pressures are 17 Psi, 20 Psi, 24 Psi and 27 Psi.

As shown in Figure 1, there is a large black background area and a faint light reflection area in each plasma image (upper and bottom parts), respectively. The plasma is mainly concentrated in the central area of the image. To explore the impact of different image areas on VOC identification, we cropped the images and created three regions covering different areas of the image (Figure 2). Region 1 (1080 × 1350 pixels, Figure S1) corresponds to the image area after cropping the image to remove the top and bottom black background areas. Initial test results showed that removing these areas had no impact on classification performance while significantly reducing the algorithm running time. Region 2 corresponds to the central area of the plasma image and region 3 is a section of the reflected light area at the bottom of the image. The region 2 and region 3 image sizes are 360 × 324 pixels and 540 × 400 pixels, respectively. Figure 3a,b show the region 2 and region 3 plasma images for the test data set. The images of the three regions are labeled according to Table 1.

**Figure 2:**
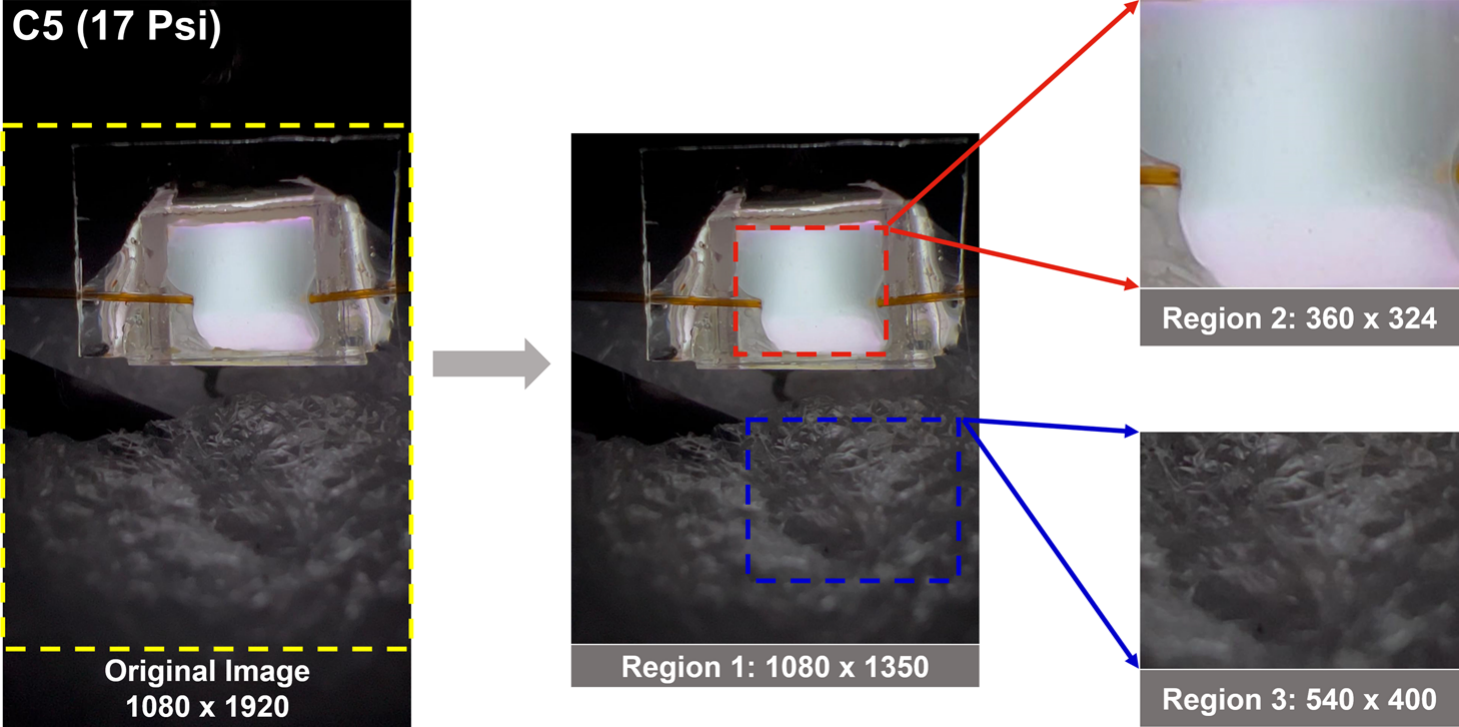
An illustration of the selection of three regions from the original plasma image. Region 1 (1080 × 1350 pixels) is the image area after removal of the top and bottom black background areas. Region 2 is a 540 × 420 pixel image enclosing the central plasma region only, and region 3 is a 540 × 400 pixels image cropped from the faint light reflection area below the plasma.

**Figure 3:**
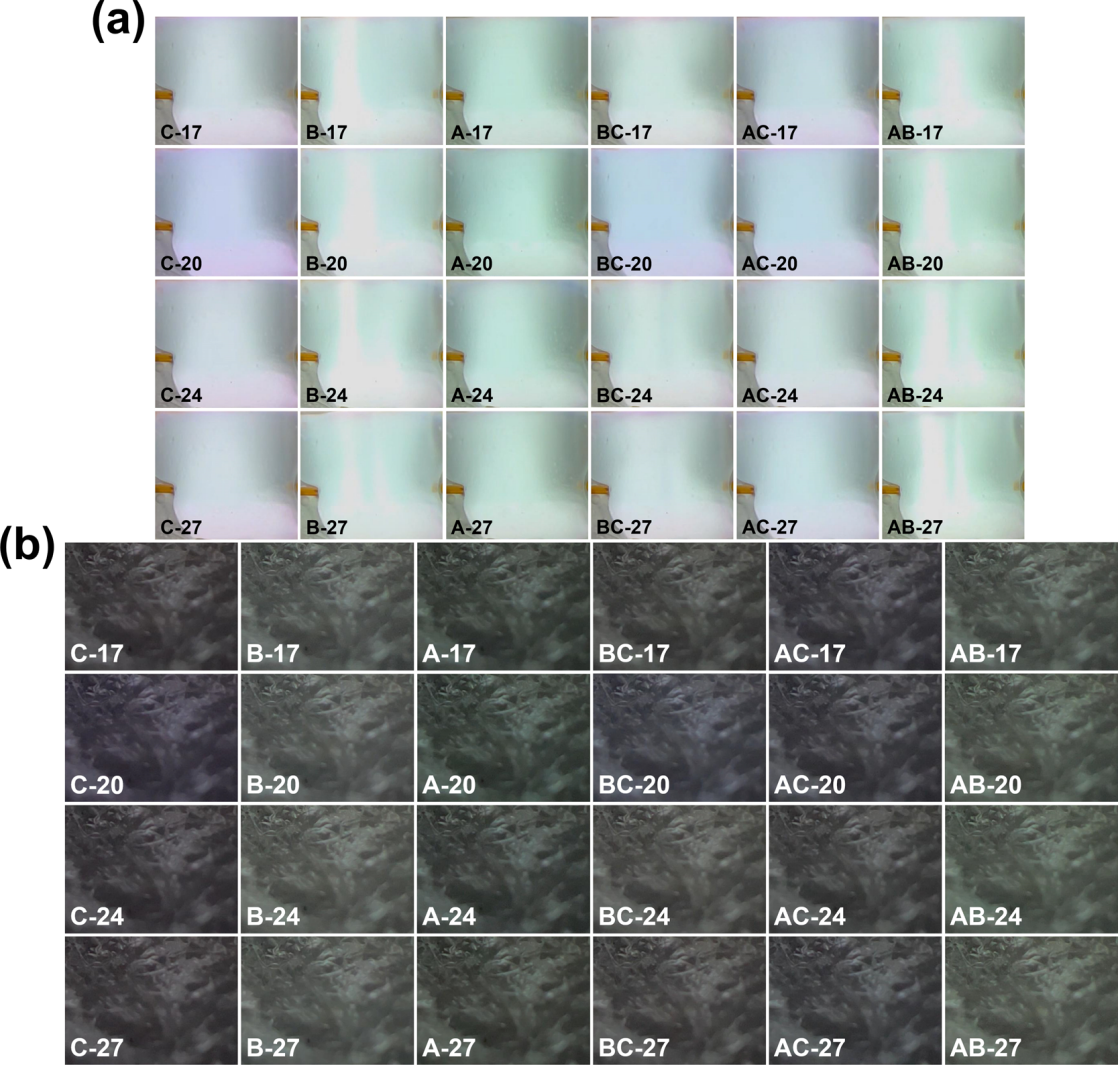
Plasma image test data sets used in this study. (a) Region 2 (360 × 324 pixels) plasma images. (b) Region 3 (540 × 400 pixels) plasma images.

### 3.2 VOC Identification Based on Plasma Image RGB Feature Vectors

The first step in image classification is to generate the characteristic feature vector corresponding to each image. Figure 4a gives the detailed overview of our approach to plasma image feature vector construction, using the plasma image of acetone injection (27 Psi) as an example. Essentially, an image is made up of a specific number of pixels, and each pixel has specific RGB values. We begin by dividing the image into segments by partitioning it in both the width and height directions to obtain a certain number of square blocks. Each block is then represented by the average RGB values of its pixels, and these values are concatenated into a one-dimensional row vector to yield the feature vector for the image. In the example in Figure 4a, the 1,458,000 pixel (1080 × 1350) region 1 image is partitioned into 80 blocks, resulting in 135 × 135 pixel blocks. This results in 80 sets of RGB average values which are concatenated into a feature vector with 240 entries (80 × 3).

**Figure 4:**
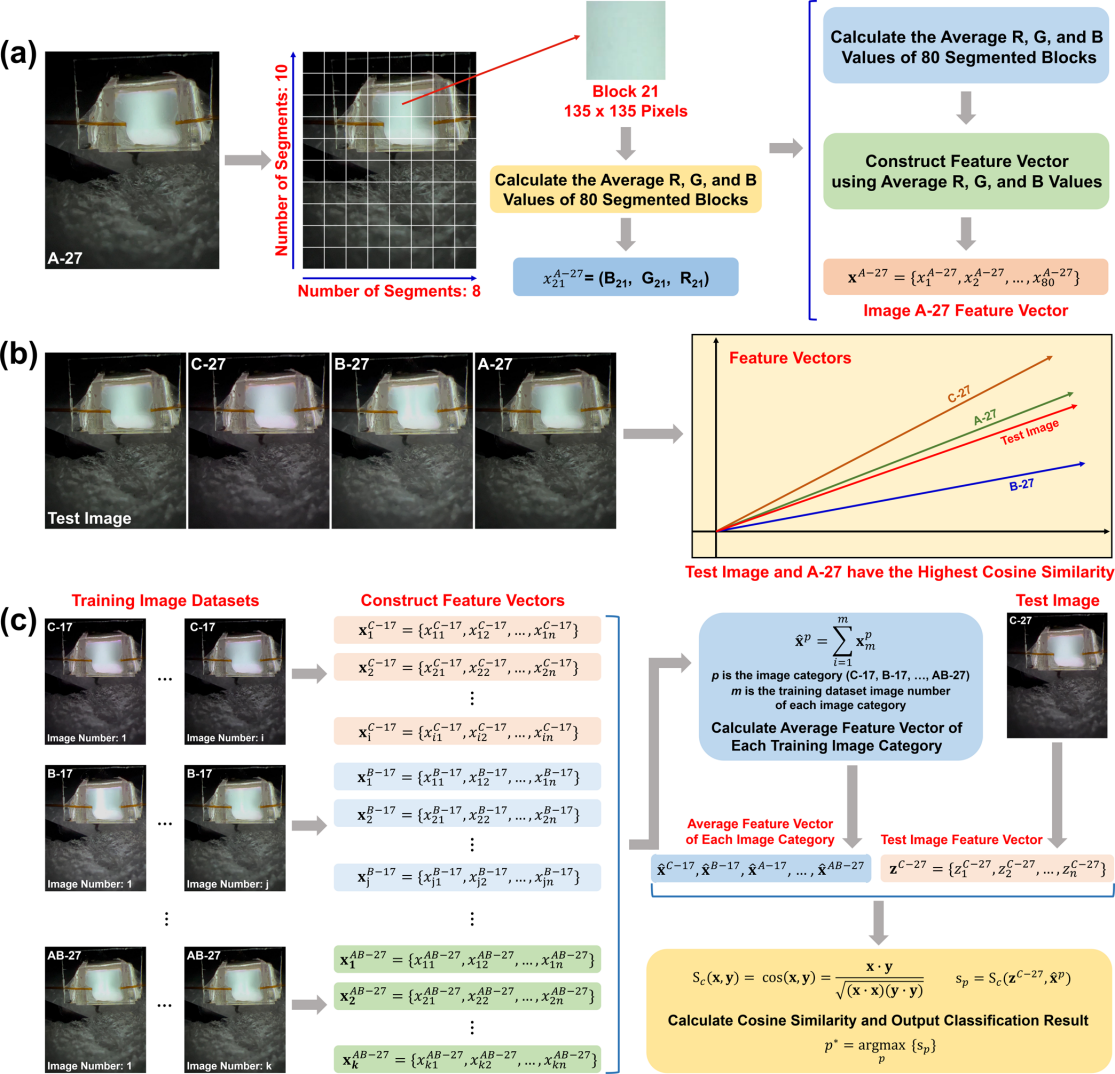
(a) Plasma image feature vector construction flowchart. (b) Plasma image recognition process based on image feature vectors (The test image here is the same as the A-27 image to facilitate demonstration). (c) VOC plasma image classification schematic diagram based on cosine similarity method (C-27 is used as the test image example here). *n* is the vector column number, which is three times the number of image segmented blocks.

VOC (image) classification is achieved by calculating the cosine value of the angle between the characteristic vectors of the test and training plasma images. The cosine similarity of two nonzero feature vectors **x** and **y** is defined as^74,75,85^

1 $$S_{c}\left(x,y\right)=\cos\left(x,y\right)=\frac{x\cdot y}{\sqrt{\left(x\cdot x\right)\left(y\cdot y\right)}}$$

Here, the value of *S*_c_(**x**,**y**) is between −1 and 1. The closer the cosine similarity value of two vectors is to 1, the more similar they are, which indicates that their corresponding plasma images are similar. Figure 4b gives an intuitive schematic diagram of classifying a test image using the cosine similarity method. The feature vector of the test image makes the smallest angle with the A-27 image feature vector, that is, it is the vector with the largest cosine similarity, hence, the test image is classified as belonging to this category.

Figure 4c presents the detailed flowchart of VOC plasma image classification based on the cosine similarity method. After inputting the test image, the algorithm calculates the cosine similarity between the test image and each image category in the training data set, and the final classification is the category of the training image that has the maximum cosine similarity value with the test image. In this study, the test data set has 24 images, and the training data set has 95 images. For the training data set, the algorithm first calculates the average characteristic feature vector of each image category using all feature vectors of that category of plasma image in the training data set (Figure 4c). Each element (column) of the average feature vector is the average value of the corresponding elements of the individual feature vectors. The algorithm then computes the cosine similarity between the test image and the 24 average feature vectors generated from the training images. Finally, the algorithm outputs the classification results based on the training image category that yields the maximum cosine similarity value.

We also explored the effect of different numbers of blocks on the image classification results. Table 2 shows the classification accuracy and the computation time corresponding to different block sizes used in this study. For all three image regions, the smallest block size is 1 × 1 pixel. Figure 5 shows samples of the original images in three regions (Figure 5a,f,k) and the corresponding reconstructed images with different block sizes. The images from the three regions transition from being blurry to clear and finally to the original images as the number of blocks increase. The algorithm classification computation time also increased rapidly since a larger number of blocks will result in the generation of higher dimension feature vectors. As shown in Table 2, the algorithm computation time when processing the maximum number of blocks for regions 1, 2, and 3 images increased by a factor of 1,310, 532, and 302, respectively compared to the test run time when processing the minimum number of blocks in each case. However, classification accuracy did not improve with increased block numbers for region 1 and region 3 images. In fact, the accuracy slightly decreased, from 100% to 95.8% for region 1 images, and from 91.7% to 87.5% for region 3 images during tests. In contrast, the classification accuracy increased from 91.7% to 95.8% as the number of blocks increased for region 2 images. All segmentation categories achieved higher classification accuracy compared to the 1 × 1 image block (Table 2). When using feature vectors constructed using the average RGB values of the entire image for computation and classification, the classification accuracies for regions 1, 2, and 3 images (1 × 1 blocks) are only 58.3%, 62.5%, and 58.3%, respectively. These results highlight the importance of considering image segmentation when using the cosine similarity method for classification.

**Table 2:** Classification Accuracy and the Associated Algorithm Computation Time of Different Segmentations Corresponding to the Number of Square Blocks for Different VOC Image Regions^a^

Region 1 (1080 × 1350 Pixels)				Region 2 (360 × 324 Pixels)				Region 3 (540 × 400 Pixels)			
number of blocks	block size	accuracy	time (s)	number of Blocks	block Size	accuracy	time (s)	number of Blocks	block Size	accuracy	time (s)
1 × 1 (1)	1080 × 1350	58.3%	3.4	1 × 1 (1)	360 × 324	62.5%	0.4	1 × 1 (1)	540 × 400	58.3%	0.6
4 × 5 (20)	270 × 270	100%	3.5	10 × 9 (90)	36 × 36	91.7%	0.7	27 × 20 (540)	20 × 20	91.7%	2.3
8 × 10 (80)	135 × 135	100%	3.8	20 × 18(360)	18 × 18	91.7%	1.6	54 × 40 (2160)	10 × 10	91.7%	7.3
12 × 15 (180)	90 × 90	95.8%	4.2	30 × 27 (810)	12 × 12	91.7%	3.0	108 × 80 (8640)	5 × 5	87.5%	27.1
20 × 25 (500)	54 × 54	100%	5.4	40 × 36 (1440)	9 × 9	95.8%	4.9	135 × 100 (13,500)	4 × 4	87.5%	42.7
24 × 30 (720)	45 × 45	100%	6.0	60 × 54 (3240)	6 × 6	95.8%	10.4	270 × 200 (54,000)	2 × 2	87.5%	190.3
36 × 45 (1620)	30 × 30	100%	8.8	90 × 81 (7290)	4 × 4	95.8%	23.2	540 × 400 (216,000)	1 × 1	87.5%	693.7
40 × 50 (2000)	27 × 27	100%	10.1	120 × 108 (12,960)	3 × 3	95.8%	40.9				
60 × 75 (4500)	18 × 18	100%	18.9	180 × 162 (29,160)	2 × 2	95.8%	92.2				
72 × 90 (6480)	15 × 15	100%	23.7	360 × 324 (116,640)	1 × 1	95.8%	372.6				
108 × 135 (14,580)	10 × 10	95.8%	49.7								
120 × 150 (18,600)	9 × 9	95.8%	62.2								
180 × 225 (40,500)	6 × 6	95.8%	133.1								
216 × 270 (58,320)	5 × 5	95.8%	192.5								
360 × 450 (162,000)	3 × 3	95.8%	530.5								
540 × 675 (364,500)	2 × 2	95.8%	1168.0								
1080 × 1350 (1458,000)	1 × 1	95.8%	4585.2								

a Image classification accuracy is calculated as the ratio of the number of correctly classified images to the total number of test images. The algorithm, implemented in python, ran on a laptop equipped with an eighth generation Intel Core i7 processor and automatically recorded the execution time. Run-times have been rounded to one decimal place.

**Figure 5:**
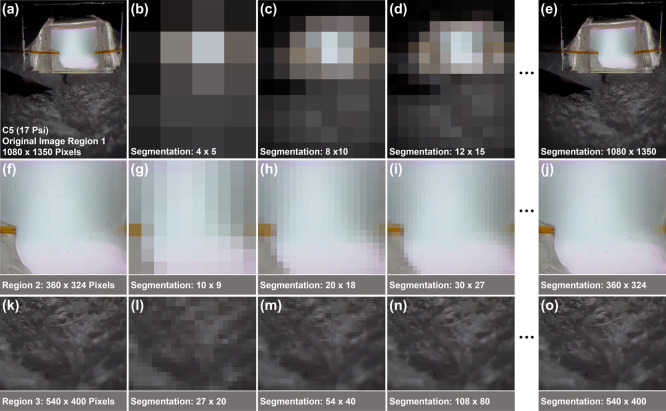
Reconstructed plasma images after segmenting the three regions of C-17 into different blocks. Images (a, f, k) are the original C-17 images before segmentation while (b–e, g–j, l–o) are the reconstructed plasma images for different numbers of segmentation blocks for region 1, region 2, and region 3, respectively.

Among the three region image tests, the region 1 image test has the highest classification accuracy (100%). However, the 100% accuracy can only be maintained when the number of blocks is relatively small (below 108 × 135 (14,580)). An exception here is the performance of 12 × 15 (180) blocks, where the classification accuracy falls to 95.8%. The only error in the test was the misclassification of B-20 as B-17. B-20 and B-17 are highly similar images, causing the model to fail. However, B-20 is still classified as the correct VOC (benzene). Region 2 images also have a high classification accuracy (95.8%), but the classification accuracy is lower for relatively small numbers of blocks (30 × 27 (810) and below). Region 2 images mainly contain the plasma and have rich and highly concentrated color information. A higher number of blocks is helpful in distinguishing region 2 image differences, which can explain the relatively low classification accuracy (91.7%) of region 2 images at lower block numbers (30 × 27 (810) and below). The classification accuracy of region 3 images is the lowest among the three regions due to the limited color information in the faint light reflection area contained within this region. For the case in which image color information is not concentrated, increasing the number of blocks is not helpful in improving the image classification accuracy.

Additionally, we also plotted the cosine similarity matrices of test results using the three image regions (Figure 6). Before plotting, we noticed that the cosine similarity values calculated by the algorithm are very close to one and the differences between the values are also very small, which means that the angles between different feature vectors are very small. Therefore, to amplify the differences for visualization purposes, we normalized the original similarity values using eq 2.

2 $$S_{norm}\left(x,y\right)=\frac{S_{c}\left(x,y\right)-S_{\min}\left(x,y\right)}{1.0-S_{\min}\left(x,y\right)}$$

Here, *S*_c_(**x**,**y**) is the cosine similarity value of the original data and S_min_(**x**,**y**) is the minimum cosine similarity value obtained in each classification test. *S*_norm_(**x**,**y**) is the normalized cosine similarity value and ranges between 0 and 1. In Figure 6, using *S*_norm_(**x**,**y**), it is easy to see that the difference in values in the cosine similarity matrices become larger as the number of blocks increases for all three region images.

**Figure 6:**
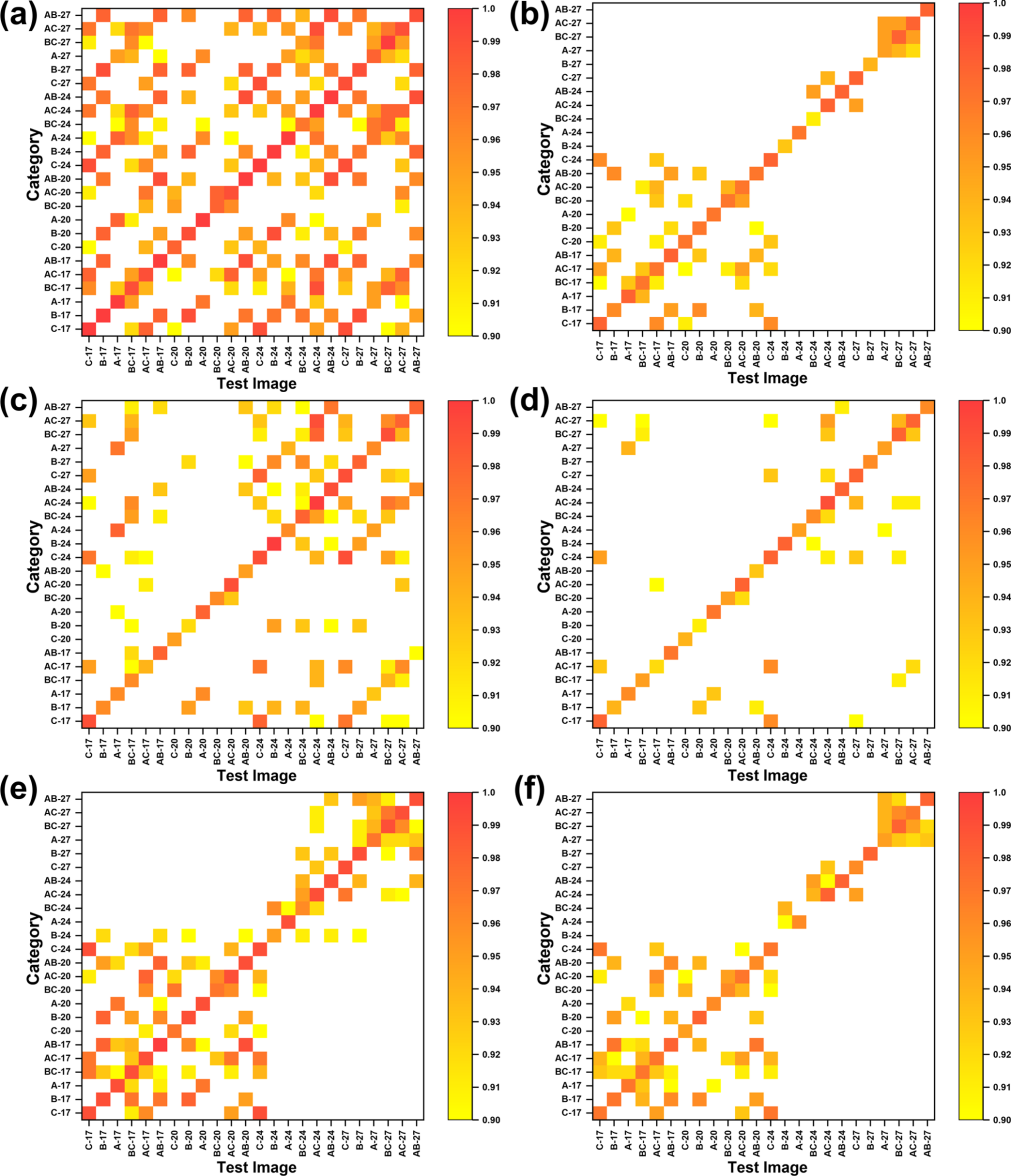
Normalized cosine similarity matrix of test results for segmenting images with different block numbers. Region 1 image: (a) 4 × 5 and (b) 1080 × 1350, Region 2 image: (c) 10 × 9 and (d) 360 × 324, Region 3 image: (e) 27 × 20 and (f) 540 × 400. Here, the normalized cosine similarity, as defined in eq 2, is calculated from the corresponding original cosine similarity values and plotted with the display lower limit set to 0.90 to highlight differences.

Table 3 shows a summary of images that were misidentified and misclassified to a different image category. Based on Table 3, the classification errors can be summarized into three types: (1) images of the same VOC/VOC mixture at different pressures, (2) images of VOC mixtures that contain the same VOC at different pressures, (3) VOC and VOC mixtures containing the aforementioned VOC at the same pressure. It is worth mentioning that no classification errors occurred for two VOCs/VOC mixtures with completely different compositions. In region 1 image tests, the algorithm misclassified images of benzene (B-20 to B-17) and VOC mixtures that contain benzene (BC-24 to AB-24). The misclassification of B-20 only occurred once when using the 90 × 90-pixel blocks as discussed above. The misclassification of BC-24 starts when the block count is 108 × 135 (14,580) and continues until the maximum block number (i.e., highest resolution). It is easy to see from Figure S1 that BC-24 and AB-24 have different colors but have similar plasma color distribution (both contain two separate light columns in the central plasma region). When the number of segmentation blocks is relatively low (14,580 and below), the color differences are mainly highlighted by the color (RGB values) homogenization of each square block, thus leading to a successful classification. When the number of small blocks is larger than 14,580, the long feature vectors generated from BC-24 and AB-24 images will become more similar due to the similarity in the plasma shape in these two images, thus resulting in misclassification. The classification errors in region 2 image tests mainly occurred between images of the same VOC at different pressures. B-20 was misclassified as category B-17 in all tests and A-17 was misclassified as A-24 with 10 × 9 segmentation or A-27 with 20 × 18 and 30 × 27 segmentation. The main reason for these misclassifications is the high similarity between these images. Region 2 images only contain the illuminated plasma area and hence the resulting feature vectors are also very similar. Therefore, region 2 images have more classification errors than region 1 images which contain faint light reflection areas. The test results of regions 1 and 2 show that the faint light reflection areas around the plasma central image contains useful RGB information. This information can be used for image classification and the region 3 image test results clearly verify the usefulness of this information. Region 3 images are cropped from the faint light reflection area at the bottom of the image but still achieve relatively high classification accuracy during tests (up to 91.7%). Unlike regions 1 and 2 images, the RGB information in region 3 is relatively small and scattered. The absence of areas with strong distinguishing features (e.g., color concentration area) makes image classification with region 3 more difficult than with regions 1 and 2. Therefore, the misclassifications in region 3 image tests include all three types of error that were summarized above. It is worth noting that benzene or benzene-containing VOC mixtures were misclassified in all region 2 and region 3 image tests. This is mainly attributed to the strong luminescence ability of benzene in μHDBD-PID. As discussed above, benzene has the lowest limit of detection and linearity range for μHDBD-PID compared to other VOCs.^60^ This results in similar plasma images when benzene is injected at different pressures and causes the plasma luminescence image of benzene mixed with other VOCs to be more similar to the plasma image of benzene injection.

**Table 3:** Images That the Algorithm Misidentified and Misclassified Results in the Tests of Different Image Regions

Region 1 (1080 × 1350 Pixels)			Region 2 (360 × 324 Pixels)			Region 3 (540 × 400 Pixels)		
number of blocks	misidentified image	misclassified category	number of blocks	misidentified image	misclassified category	number of blocks	misidentified image	misclassified category
12 × 15 (180)	B-20	B-17	10 × 9 (90)	A-17	A-24	27 × 20 (540)	C-24	C-17
				B-20	B-17		B-24	BC-24
108 × 135 (14,580)	BC-24	AB-24	20 × 18 (360)	A-17	A-27	54 × 40 (2160)	B-24	BC-24
				B-20	B-17		BC-24	AC-24
120 × 150 (18,600)	BC-24	AB-24	30 × 27 (810)	A-17	A-27	108 × 80 (8640)	AB-20	AB-17
				B-20	B-17		B-24	BC-24
							BC-24	AB-24
180 × 225 (40,500)	BC-24	AB-24	40 × 36 (1440)	B-20	B-17	135 × 100 (13,500)	AB-20	AB-17
							B-24	BC-24
							BC-24	AB-24
216 × 270 (58,320)	BC-24	AB-24	60 × 54 (3240)	B-20	B-17	270 × 200 (54,000)	AB-20	AB-17
							B-24	BC-24
							BC-24	AB-24
360 × 450 (162,000)	BC-24	AB-24	90 × 81 (7290)	B-20	B-17	540 × 400 (216,000)	AB-20	AB-17
							B-24	BC-24
							BC-24	AB-24
540 × 675 (364,500)	BC-24	AB-24	120 × 108 (12,960)	B-20	B-17			
1080 × 1350 (1458,000)	BC-24	AB-24	180 × 162 (29,160)	B-20	B-17			
			360 × 324 (116,640)	B-20	B-17			

The classification tests of region 1, 2, and 3 images demonstrated the impact of image areas on classification accuracy. Images of the plasma area only (region 2) or images with reflected information only (region 3) may not be the best options to achieve optimal classification performance. In contrast, region 1 images with the composite image content (combination of regions 2 and 3) achieved the best classification performance (100%). These results also indicate that segmenting the images into blocks to obtain a richer feature vector for classification is beneficial, but having a high level of segmentation (i.e., a large number of blocks) is not necessarily beneficial. Table S2 summarizes the image misclassifications and their frequency of occurrence for each region. There are a total of eight image misclassifications, and their frequencies vary across the three regions. Region 1 images produce the fewest misclassifications (two) while region 3 images produce the most (five). Moreover, the misclassification categories in regions 2 and 3 are complementary. We also conducted tests using the concatenated feature vectors from region 2 and 3 images, and achieved a classification accuracy of 95.8% or better for different block size combinations. One combination, region 2 (10 × 9) + region 3 (27 × 20), achieved 100% classification accuracy. These results further demonstrate that using images with composite information (as is the case with region 1) improves classification performance.

Helium plasma images without VOC injection also have differences at different GC inlet pressures. We selected images of helium plasma at four different test pressures and indexed them as He-17, He-20, He-24, and He-27 (Table S3). We selected seven images of each type of helium plasma - one as the test image and six as training images. Figure S2 presents the original helium plasma images at the different GC inlet pressures and the helium plasma test images for the three regions. As shown in Figure S2a, the color of the helium plasma shifts from lilac to white as the GC inlet pressure increases. It is easy to see that the differences between the helium plasma at four different pressures are very minor, but our algorithm can still classify these images with 100% accuracy using all three image regions. The helium plasma image classification results and algorithm computation time are summarized in Table S4. Helium plasma images achieved 100% classification accuracy across all three regions. This high accuracy compared to VOC images stems from the strong similarity between helium plasma images at the same pressure. Tables S5 and S6 present the calculated mean, maximum, and difference (maximum - mean) of cosine similarity between all VOC/helium plasma images (test and training data sets) of each category and their average feature vectors. These calculations are performed using regions 1, 2, and 3 images from the same segmentation. Region 1 is 4 × 5, region 2 is 40 × 36, and region 3 is 27 × 20. The calculated differences for helium plasma images are 1–2 orders of magnitude smaller than those for VOCs images. This indicates a much higher degree of similarity for helium plasma images than VOC images. Consequently, the average feature vector for each training helium plasma image category is always very similar to the test plasma image feature vector. As a result, the test images consistently have the highest cosine similarity with their corresponding average feature vectors during validation. This explains the 100% classification accuracy achieved across all segmentations and resolutions.

The 100% classification accuracy with region 3 images also confirms that the subtle variations in helium plasma with inlet pressure are also captured in the faint light reflection areas of the image. Additionally, the algorithm computation time is greatly reduced due to the relatively small image data set size compared to the VOC/VOC mixture images (Table S4). Compared with the VOC/VOC mixture plasma image classification results, helium plasma classification test results demonstrate the importance of stable training data sets in achieving excellent image classification performance.

The classification test results for VOC/VOC mixture and helium plasma images show that increasing the number of blocks may not be the best option for improving classification accuracy. Besides, increasing the number of blocks also significantly increases computational complexity and hence the algorithm run time. The tests using the concatenated feature vectors of regions 2 and 3 images clearly verify that the difference in the image content of each region is the main reason for the different classification results. The classification tests of helium plasma images also confirmed the importance of stable image data sets for high classification accuracy.

## 4 Conclusion

In this paper, we propose an RGB image-based VOC identification method. This new method identifies VOCs by calculating the cosine similarity between characteristic feature vectors of images of the plasma recorded during VOC injection into the μHDBD-PID. The characteristic feature vectors are constructed using the image RGB values. The cosine similarity based algorithm achieved 100% classification accuracy for region 1 image tests and over 90% accuracy with features extracted from two image subregions (denoted region 2 and 3). Our study also shows the impact of image content and the level of segmentation (i.e., number of segmentation blocks) on classification performance. The diversity of image content and the high similarity between the test and the training data set images are conducive to achieving high classification accuracy. Overall, the proposed cosine similarity-based assessment of RGB feature vectors enables a simple and easy-to-operate approach for VOC identification without the need for data-intensive model training.

## Supplementary Material



## Data Availability

Test and training data set plasma images (ZIP): https://zenodo.org/records/20099619.

## Notes

Comparison of different detection techniques used for VOCs (Table S1); region 1 plasma image test data set (Figure S1); image misclassification type and frequency of occurrence in each region image test (Table S2); helium plasma image allocated identifiers at four different pressure values (Table S3); original helium plasma images and test helium plasma images in different regions (Figure S2); helium plasma image classification accuracy and the associated program computation time of different segmentations (Table S4); mean, maximum, and difference (maximum - mean) of the cosine similarity between VOC images (test and training data sets) of each category and their average feature vector for images of three regions (Table S5); mean, maximum, and difference (maximum - mean) of the cosine similarity between helium plasma images (test and training data sets) of each category and their average feature vector for images of three regions (Table S6); and python code for VOCs identification using the cosine similarity method (PDF)

## References

[1] Khatib M., Haick H. (2022). Sensors for volatile organic compounds. ACS
Nano.

[2] Ye Q., Chen Y., Li Y., Jin R., Geng Q., Chen S. (2023). Management of typical VOCs in air
with adsorbents: status and challenges. Dalton
Trans..

[3] Li A. J., Pal V. K., Kannan K. (2021). A review of
environmental occurrence,
toxicity, biotransformation and biomonitoring of volatile organic
compounds. Environ. Chem. Ecotoxicol..

[4] Mangotra A., Singh S. K. (2024). Volatile organic
compounds: A threat to the environment
and health hazards to Living Organisms-A Review. J. Biotechnol..

[5] Helmig D. (1999). Air analysis
by gas chromatography. J. Chromatogr. A.

[6] Dewulf J., Van Langenhove H., Wittmann G. (2002). Analysis of volatile organic compounds
using gas chromatography. TrAC, Trends Anal.
Chem..

[7] Lim Y. M., Swamy V., Ramakrishnan N., Chan E. S., Kesuma H. P. (2023). Volatile
organic compounds (VOCs) in wastewater: Recent advances in detection
and quantification. Microchem. J..

[8] Langford V. S., Graves I., McEwan M. J. (2014). Rapid monitoring
of volatile organic
compounds: a comparison between gas chromatography/mass spectrometry
and selected ion flow tube mass spectrometry. Rapid Commun. Mass Spectrom..

[9] Majchrzak T., Wojnowski W., Lubinska-Szczygeł M., Różańska A., Namieśnik J., Dymerski T. (2018). PTR-MS and GC-MS as complementary
techniques for analysis of volatiles: A tutorial review. Anal. Chim. Acta.

[10] Schulz E., Woollam M., Vashistha S., Agarwal M. (2024). Quantifying exhaled
acetone and isoprene through solid phase microextraction and gas chromatography-mass
spectrometry. Anal. Chim. Acta.

[11] Shakeel H., Rice G. W., Agah M. (2014). Semipacked columns
with atomic layer-deposited
alumina as a stationary phase. Sens. Actuators,
B.

[12] Epping R., Koch M. (2023). On-site detection of
volatile organic compounds (VOCs). Molecules.

[13] Lin Z., Abbott J., Karuso P., Wong D. K. (2025). Advances in electroanalytical
sensing of volatile organic compounds towards field-deployable detection. TrAC, Trends Anal. Chem..

[14] Tian Y., Liu Y., Dong K., Zhao B., Tang S., Nie X., Yan Y. (2025). Advances in
rapid detection of volatile organic compounds (VOCs):
From conventional techniques to surface-enhanced Raman spectroscopy. Results Chem..

[15] Kanan S., Obeideen K., Moyet M., Abed H., Khan D., Shabnam A., El-Sayed Y., Arooj M., Mohamed A. A. (2025). Recent
advances on metal oxide based sensors for environmental gas pollutants
detection. Crit. Rev. Anal. Chem..

[16] Guo X., Shen J., Liu Z., Guo B. B., Love D., McKinney P. J., Zhang J. (2026). Experimental
Evaluation of Low-Cost
Metal Oxide Volatile Organic Compounds Sensors for Indoor Air Quality
Monitoring. Build. Environ..

[17] Liu K., Zhang C. (2021). Volatile organic
compounds gas sensor based on quartz crystal microbalance
for fruit freshness detection: A review. Food
Chem..

[18] Pérez R. L., Ayala C. E., Park J.-Y., Choi J.-W., Warner I. M. (2021). Coating-based
quartz crystal microbalance detection methods of environmentally relevant
volatile organic compounds. Chemosensors.

[19] Cao Y., Fu M., Fan S., Gao C., Ma Z., Hou D. (2024). Hydrophobic
MOF/PDMS-based QCM sensors for VOCs identification and quantitative
detection in high-humidity environments. ACS
Appl. Mater. Interfaces.

[20] Li X., Sun W., Fu W., Lv H., Zu X., Guo Y., Gibson D., Fu Y.-Q. (2023). Advances in sensing mechanisms and
micro/nanostructured sensing layers for surface acoustic wave-based
gas sensors. J. Mater. Chem. A.

[21] Zhou H., Ramaraj S. G., Sarker M. S., Tang S., Yamahara H., Tabata H. (2025). Parts-per-trillion-level
acetone gas detection using
a suspended graphene/SiO_2_ SAW breath and skin gas sensor:
Simulation and experimental study. ACS Sens..

[22] Rezende G. C., Le Calvé S., Brandner J. J., Newport D. (2019). Micro photoionization
detectors. Sens. Actuators, B.

[23] Cai Y., Che X., Duan Y. (2025). From Volume to Mass: Transforming
Volatile Organic
Compound Detection with Photoionization Detectors and Machine Learning. Sensors.

[24] Li Y., Wang Z., Zhao T., Li H., Jiang J., Ye J. (2024). Electronic nose for the detection
and discrimination of volatile
organic compounds: Application, challenges, and perspectives. TrAC, Trends Anal. Chem..

[25] He S., Wen J., Cao B., Shi G., Zhang M. (2025). Porous Material-Based
Electronic Noses for the Sensing of Volatile Organic Compounds. ACS Appl. Mater. Interfaces.

[26] Hernik A., Smets J., Wang Z., Semenova Y., Tan J. C., Ameloot R., Naydenova I. (2025). Optical detection
of volatile organic
compounds: a review of methods and functionalized sensing materials. Adv. Opt. Mater..

[27] Lee Y., Son H., Lee J., Lim S.-H. (2024). Review on micro-gas chromatography
system for analysis of multiple low-concentration volatile organic
compounds: preconcentration, separation, detection, integration, and
challenges. Micro Nano Syst. Lett..

[28] Lee Y., Lee S., Jang W., Lee J., Choi Y., Lim S.-H. (2025). Hybrid
GC platform: a micro gas chromatography system with a simple configuration
for low-concentration VOC analysis. Lab Chip.

[29] Thamatam N., Chowdhury M., Agah M. (2025). Fluidic and Electrical Modular Interfacing:
A modular approach to micro total analytical systems and micro gas
chromatography. Sens. Actuators, B.

[30] Duan C., Li J., Zhang Y., Ding K., Geng X., Guan Y. (2022). Portable instruments
for on-site analysis of environmental samples. TrAC, Trends Anal. Chem..

[31] Crucello J., de Oliveira A. M., Sampaio N. M. F. M., Hantao L. W. (2022). Miniaturized systems
for gas chromatography: Developments in sample preparation and instrumentation. J. Chromatogr. A.

[32] Chowdhury M., Manurkar S., Rege V., Gupta P., Sharma A., Nazhandali L., Agah M. (2023). Miniaturized gas chromatographic
nose for on-site adulteration detection. IEEE
Trans. Instrum. Meas..

[33] Regmi B. P., Agah M. (2018). Micro gas chromatography:
An overview of critical components and
their integration. Anal. Chem..

[34] Li M. W.-H., Ghosh A., Sharma R., Zhu H., Fan X. (2021). Integrated
microfluidic helium discharge photoionization detectors. Sens. Actuators, B.

[35] Wei-Hao
Li M., Ghosh A., Venkatasubramanian A., Sharma R., Huang X., Fan X. (2021). High-sensitivity micro-gas chromatograph-photoionization detector
for trace vapor detection. ACS Sens..

[36] Cruz D., Chang J., Showalter S., Gelbard F., Manginell R., Blain M. (2007). Microfabricated thermal
conductivity detector for the micro-ChemLab. Sens. Actuators, B.

[37] Akbar M., Narayanan S., Restaino M., Agah M. (2014). A purge and
trap integrated
microGC platform for chemical identification in aqueous samples. Analyst.

[38] Sun J., Cui D., Chen X., Zhang L., Cai H., Li H. (2013). A micro gas
chromatography column with a micro thermal conductivity detector for
volatile organic compound analysis. Rev. Sci.
Instrum..

[39] Rizk-Bigourd M., Szopa C., Coscia D., Pineau J.-P., Guerrini V., Ferreira F., Bertrand F., Philippart A., Boco A., Rioland G. (2024). Development
and Integration
of an Ultraminiaturized Gas Chromatograph Prototype Based on Lab-on-a-Chip
Microelectromechanical Systems for Space Exploration Missions. ACS Earth Space Chem..

[40] Kuipers W., Müller J. (2010). Sensitivity
of a planar micro-flame ionization detector. Talanta.

[41] Kuipers W., Müller J. (2011). Characterization of a microelectromechanical systems-based
counter-current flame ionization detector. J.
Chromatogr. A.

[42] Raut R. P., Thurbide K. B. (2017). Characterization of titanium tiles
as novel platforms
for micro-flame ionization detection in miniature gas chromatography. Chromatographia.

[43] Sukaew T., Chang H., Serrano G., Zellers E. T. (2011). Multi-stage preconcentrator/focuser
module designed to enable trace level determinations of trichloroethylene
in indoor air with a microfabricated gas chromatograph. Analyst.

[44] von
Mühlen C., Khummueng W., Alcaraz Zini C., Bastos Caramão E., Marriott P. J. (2006). Detector technologies
for comprehensive two-dimensional gas chromatography. J. Sep. Sci..

[45] Qin Y., Gianchandani Y. B. (2016). A fully electronic microfabricated gas chromatograph
with complementary capacitive detectors for indoor pollutants. Microsyst. Nanoeng..

[46] Liao W., Winship D., Lara-Ibeas I., Zhao X., Xu Q., Lu H.-T., Qian T., Gordenker R., Qin Y., Gianchandani Y. B. (2023). Highly
integrated μGC based
on a multisensing progressive cellular architecture with a valveless
sample inlet. Anal. Chem..

[47] Xu Q., Zhao X., Qin Y., Gianchandani Y. B. (2024). Control
Software Design for a Multisensing Multicellular Microscale Gas Chromatography
System. Micromachines.

[48] Wang J., Bryant-Genevier J., Nuñovero N., Zhang C., Kraay B., Zhan C., Scholten K., Nidetz R., Buggaveeti S., Zellers E. T. (2018). Compact prototype microfabricated gas chromatographic
analyzer for autonomous determinations of VOC mixtures at typical
workplace concentrations. Microsyst. Nanoeng..

[49] Kim S. K., Chang H., Zellers E. T. (2011). Microfabricated
gas chromatograph
for the selective determination of trichloroethylene vapor at sub-parts-per-billion
concentrations in complex mixtures. Anal. Chem..

[50] Wang J., Nuñovero N., Nidetz R., Peterson S. J., Brookover B. M., Steinecker W. H., Zellers E. T. (2019). Belt-mounted micro-gas-chromatograph
prototype for determining personal exposures to volatile-organic-compound
mixture components. Anal. Chem..

[51] Mu X., Covington E., Rairigh D., Kurdak C., Zellers E., Mason A. J. (2012). CMOS monolithic
nanoparticle-coated chemiresistor array
for micro-scale gas chromatography. IEEE Sens.
J..

[52] Lewis P. R., Manginell P., Adkins D. R., Kottenstette R. J., Wheeler D. R., Sokolowski S. S., Trudell D. E., Byrnes J. E., Okandan M., Bauer J. M. (2006). Recent advancements
in the gas-phase MicroChemLab. IEEE Sens. J..

[53] Lu C. J., Jin C., Zellers E. T. (2006). Chamber
evaluation of a portable GC with tunable retention
and microsensor-array detection for indoor air quality monitoring. J. Environ. Monit..

[54] Hu, J. ; Qu, H. ; Guo, W. ; Chang, Y. ; Pang, W. ; Duan, X. Film bulk acoustic wave resonator for trace chemical warfare agents simulants detection in micro chromatography. In 20th International Conference on Solid-State Sensors, Actuators and Microsystems & Eurosensors XXXIII (TRANSDUCERS & EUROSENSORS XXXIII); IEEE: Piscataway, NJ, 2019; pp 45–48.

[55] Wang, Z. ; Sun, X. ; Chang, Y. ; Duan, X. ; Pang, W. In FBAR Gas Sensors; Lieberzeit, P. , Ed.; FBAR Gas Sensors, Springer Series on Chemical Sensors and Biosensors; Springer, 2023; Vol. 18.

[56] Shopova S. I., White I. M., Sun Y., Zhu H., Fan X., Frye-Mason G., Thompson A., Ja S.-j. (2008). On-column
micro
gas chromatography detection with capillary-based optical ring resonators. Anal. Chem..

[57] Collin W. R., Scholten K. W., Fan X., Paul D., Kurabayashi K., Zellers E. T. (2016). Polymer-coated micro-optofluidic ring resonator detector
for a comprehensive two-dimensional gas chromatographic microsystem:
μGC × μGC-μOFRR. Analyst.

[58] Li M., Myers E., Tang H., Aldridge S., McCaig H., Whiting J., Simonson R., Lewis N., Roukes M. (2010). Nanoelectromechanical
resonator arrays for ultrafast, gas-phase chromatographic chemical
analysis. Nano Lett..

[59] Martin O., Gouttenoire V., Villard P., Arcamone J., Petitjean M., Billiot G., Philippe J., Puget P., Andreucci P., Ricoul F. (2014). Modeling and design of a fully integrated gas analyzer
using a μGC and NEMS sensors. Sens. Actuators,
B.

[60] Mao J., Liu L., Atwa Y., Hou J., Wu Z., Shakeel H. (2023). Colorimetric
signal readout for the detection of volatile organic compounds using
a printable glass-based dielectric barrier discharge-type helium plasma
detector. ACS Meas. Sci. Au.

[61] Mao J., Atwa Y., Wu Z., McNeill D., Shakeel H. (2024). Identification
of different classes of VOCs based on optical emission spectra using
a dielectric barrier helium plasma coupled with a mini spectrometer. ACS Meas. Sci. Au.

[62] Chen C., Isa N. A. M., Liu X. (2025). A review of
convolutional neural
network based methods for medical image classification. Comput. Biol. Med..

[63] Kaur R., Kumar R., Gupta M. (2023). Deep neural
network for food image
classification and nutrient identification: A systematic review. Rev. Endocr. Metab. Disord..

[64] Wäldchen J., Mäder P. (2018). Plant species identification using
computer vision
techniques: a systematic literature review. Arch. Comput. Methods Eng..

[65] Gao Y., Xue X., Qin G., Li K., Liu J., Zhang Y., Li X. (2024). Application of machine
learning in automatic image identification
of insects-a review. Ecol. Inform..

[66] Paheding S., Saleem A., Siddiqui M. F. H., Rawashdeh N., Essa A., Reyes A. A. (2024). Advancing horizons in remote sensing:
a comprehensive survey of deep learning models and applications in
image classification and beyond. Neural Comput.
Appl..

[67] Tan S. H., Chuah J. H., Chow C.-O., Kanesan J., Leong H. Y. (2024). Artificial
intelligent systems for vehicle classification: A survey. Eng. Appl. Artif. Intell..

[68] Yang Y., Yang S., Li C., Yu Z. (2021). Recognition of plasma
discharge patterns based on CNN and visible images. IEEE Access.

[69] Yu, Z. ; Yang, S. ; Li, C. ; Yang, Y. Plasma real-time diagnostics based on visual image recognition. In 2021 IEEE 4th International Electrical and Energy Conference (CIEEC), IEEE, 2021; Wuhan, China, pp 1–4.

[70] Bong C., Kim B. S., Ali M. H., Kim D., Bak M. S. (2023). Machine
learning-based prediction of operation conditions from plasma plume
images of atmospheric-pressure plasma reactors. J. Phys. D: Appl. Phys..

[71] Li B., Chen W., Bian S., Lusi A., Tang X., Liu Y., Guo J., Zhang D., Yang C., Huang F. (2024). Recognition
of ethylene plasma image based on dual residual with attention mechanism
network. Rend. Lincei, Sci. Fis. Nat..

[72] Harris S. B., Rouleau C. M., Xiao K., Vasudevan R. K. (2024). Deep learning
with plasma plume image sequences for anomaly detection and prediction
of growth kinetics during pulsed laser deposition. npj Comput. Mater..

[73] Yin X., Song Q., Cheng S., Zhang H. (2024). Deep learning via CNN
for identification of blue core phenomenon in helicon plasma discharge. Phys. Plasmas.

[74] Falato M.
J., Wolfe B. T., Natan T. M., Zhang X., Marshall R. S., Zhou Y., Bellan P. M., Wang Z. (2022). Plasma image classification
using cosine similarity constrained convolutional neural network. J. Plasma Phys..

[75] Xia P., Zhang L., Li F. (2015). Learning similarity with cosine similarity
ensemble. Inf. Sci..

[76] Alewiwi M., Orencik C., Savaş E. (2016). Efficient
top-k similarity document
search utilizing distributed file systems and cosine similarity. Clust. Comput..

[77] Soyusiawaty, D. ; Zakaria, Y. Book data content similarity detector with cosine similarity (case study on digilib.uad.ac.id). In 12th International Conference on Telecommunication Systems, Services, and Applications (TSSA); IEEE: Piscataway, NJ, 2018; pp 1–6.

[78] Park K., Hong J. S., Kim W. (2020). A methodology
combining cosine similarity
with classifier for text classification. Appl.
Artif. Intell..

[79] Li, B. ; Han, L. Distance weighted cosine similarity measure for text classification. In Intelligent Data Engineering and Automated Learning - IDEAL 2013:14th International Conference, IDEAL 2013, Hefei, China, October 20–23, 2013. Proceedings 14. Lecture Notes in Computer Science (LNISA); Springer: Berlin, 2013; Vol. 8206, pp 611–618.

[80] Nyein, S. S. Mining contents in Web page using cosine similarity. 2011 3rd International Conference on Computer Research and Development, 2011, Vol. 2, pp 472–475.

[81] Jiang Y., Huang D., Zhang H., Jiang T., Xu W. (2023). Smart miniature
mass spectrometer enabled by machine learning. Anal. Chem..

[82] Deng F., Zhao Z., Wang R., Xiang C., Lv Y., Li W., Duan Y. (2023). Rapid and Online Detection of Foodborne Bacteria via
a Novel Ultraviolet Photoionization Time-of-Flight Mass Spectrometry. J. Agric. Food Chem..

[83] Aghili N. S., Rasekh M., Karami H., Edriss O., Wilson A. D., Ramos J. (2023). Aromatic fingerprints: VOC analysis
with E-nose and GC-MS for rapid
detection of adulteration in sesame oil. Sensors.

[84] Fathimah R. N., Majchrzak T. (2024). Investigation of the Frying Fume Composition During
Deep Frying of Tempeh Using GC-MS and PTR-MS. Molecules.

[85] Zhu X., Su S., Fu M., Liu J., Zhu L., Yang W., Jing G., Guo Y. (2018). A cosine similarity
algorithm method
for fast and accurate monitoring of dynamic droplet generation processes. Sci. Rep..

