# Supplementary material for: Image-Based Volatile Organic Compound Identification Using the Cosine Similarity Method
Source: ACS Omega. 2026 May 19;11(21):31013–26. doi: 10.1021/acsomega.6c00450 (PMC13234660; doi:10.1021/acsomega.6c00450)
Supplement: Supplementary file 1 [file ao6c00450_si_001.pdf]

Supporting Information

Image-based Volatile Organic Compound Identification using the Cosine Similarity Method

Jingqin Mao[a], Zhenxun Wu[b], Seán McLoone[a], and Hamza Shakeel[a*]

Jingqin Mao, Seán McLoone, Hamza Shakeel

a School of Electronics, Electrical Engineering and Computer Science, Queen's University Belfast, Belfast, BT7 1NN, United Kingdom

Zhenxun Wu

b Department of Science, Technology, Engineering and Public Policy, University College London, Gower Street, London, WC1E 6BT, United Kingdom

* Corresponding Author: Hamza Shakeel. Email address: H.Shakeel@qub.ac.uk

Keywords: volatile organic compounds, dielectric barrier helium plasma, photoionization detectors, image recognition, cosine similarity method, micro gas chromatography

Table S1. Comparison of different detection techniques used for VOCs

Technology	Working Principle	Advantages	Disadvantages	References
Electrochemical Sensors (Voltage, Current, and Impedance)	Measure electrical signal generated by redox reactions between VOCs and electrodes	Miniaturized, low power consumption, suitable for on-site deployment, and selective (achieved by specific design)	Sensitive to humidity, signal drift over time, and poor long-term stability	1, 2
Metal Oxide Sensors	Measure metal oxide resistance and conductivity changes caused by VOC adsorption on surface	Low-cost, small size, low limit of detection (LoD) (ppb - ppm), and wide detection range	High power consumption, long measurement time, cross-sensitivity to some inorganic gases and easily affected by temperature and humidity	3, 4
Quartz Crystal Microbalance (QCM) Sensors	Utilize piezoelectric effect of quartz crystal resonators and uses Sauerbrey equation to calculate the linear relationship between change in resonance frequency and mass of adsorbed gas	Low cost, simple structure, tunable sensing characteristics (by changing coating materials)	Poor LoD performance, easily affected by temperature and humidity, long response and recovery times, and poor long-term stability	5-7
Surface acoustic wave (SAW) Sensors	Utilize the changes in acoustic wave propagation characteristics on piezoelectric substrates induced by adsorption of VOCs	Rapid response, high sensitivity, wireless, and non-contact operation	High cost, easily affected by humidity, limited selectivity, complex signal processing, and poor sensing material stability	8, 9
Photoionization Detectors (PIDs)	PIDs utilize UV light to ionize VOCs and output current signal produced by the charge transfer of generated ions at the electrode surface	Small size, lightweight, high sensitivity, and rapid response	Can only detect total concentration of VOCs and lacks selectivity, unable to detect VOCs with high ionization potential (higher than the UV photon energy)	10, 11
Electronic Nose	Utilize sensor arrays and pattern recognition algorithms to detect and identify VOCs	Can achieve specific VOCs recognition by analyzing the "fingerprint spectrum" of VOCs	Limited sensitivity and selectivity, easily affected by humidity and temperature, baseline drift, lack of standardization in algorithms, high cost, and difficult to miniaturize	12, 13
Optical Sensors (Absorption, Scattering, Diffraction, Reflection, Refraction, and Luminescence)	Utilize the changes in wavelength, light intensity, or polarization caused by the interactions between light (UV, visible, or infrared regions) and matter for detection and identification of VOCs	Ease of operation, rapid response, immunity to electromagnetic fields, can be deployed on-site, and resistant to temperature and humidity	High cost (especially the tunable optical sources), large and difficult to miniaturize	14, 15
Micro Gas Chromatography (μGC) Systems	Same as the traditional benchtop GC systems, all components are miniaturized (e.g., micro gas preconcentrator, micro GC column, mini/micro-detector, pump, and valve)	Portable, cost effective, energy-efficient, can conduct on-site and real-time VOCs analysis	High price (compared with other types of portable VOC sensors), operational reliability (especially in harsh humidity and temperature conditions) and multispecies VOCs detection requires further improvement	16-18

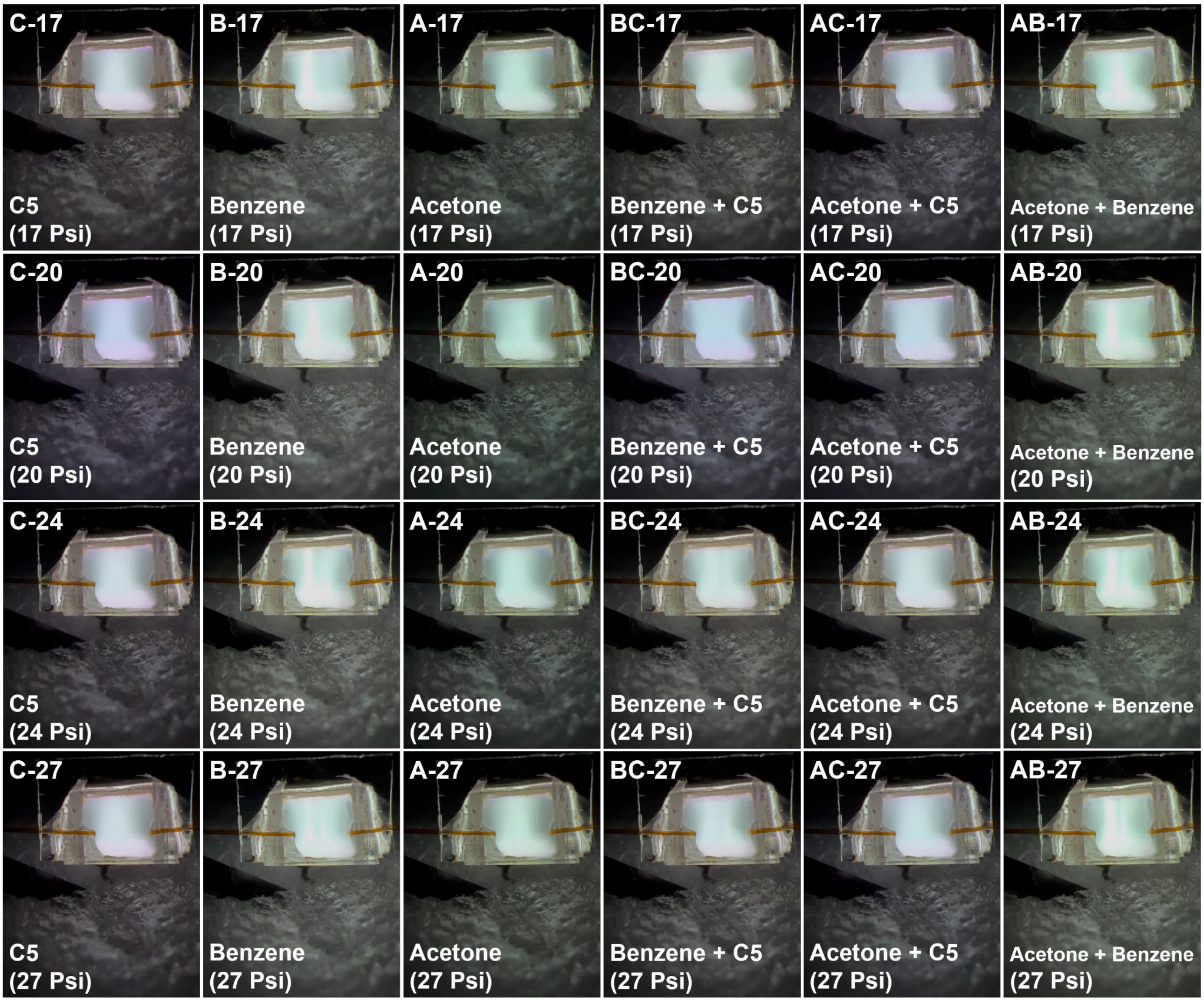

Figure S1. Region 1 plasma image test dataset.

Table S2. Type of image misclassification and frequency of occurrence in each region image test.

No.	Misclassification	Frequency of Occurrence During Tests		
		Region 1	Region 2	Region 3
1	B-20 → B-17	1	9	0
2	BC-24 → AB-24	7	0	4
3	BC-24 → AC-24	0	0	1
4	A-17 → A-24	0	1	0
5	A-17 → A27	0	2	0
6	C-24 → C-17	0	0	1
7	B-24 → BC-24	0	0	6
8	AB-20 → AB-17	0	0	4

Table S3. Allocated helium plasma image identifiers at four different pressure values.

	17 Psi	20 Psi	24 Psi	27 Psi
Helium Plasma	He-17	He-20	He-24	He-27

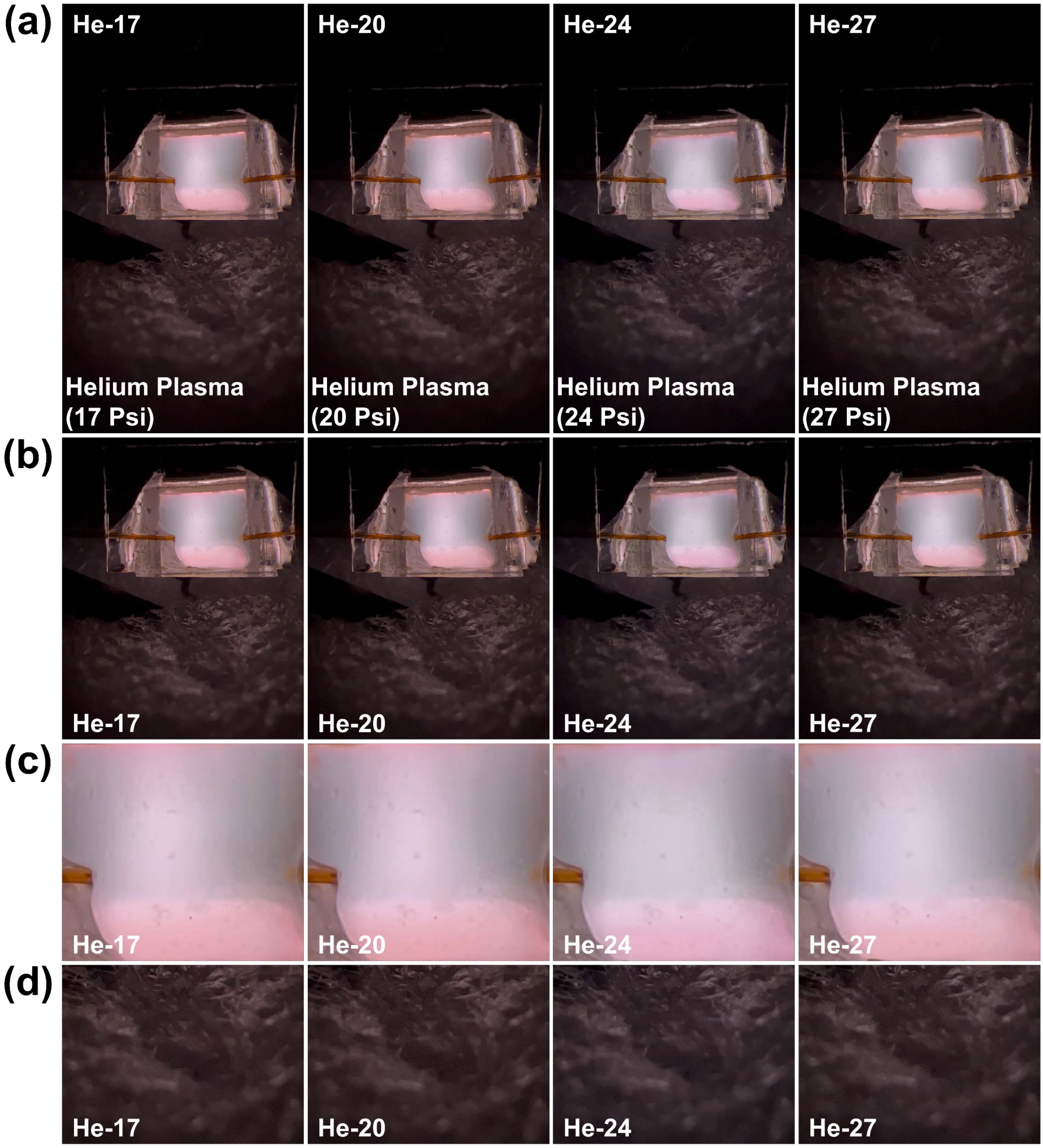

Figure S2. (a) Original helium plasma images at different GC inlet pressures (17, 20, 24, 27 Psi). (b) Region 1 helium plasma test images. (c) Region 2 helium plasma test images. (d) Region 3 helium plasma test images.

Table S4. Helium plasma image classification accuracy and the associated program computation time of different segmentations corresponding to the number of square blocks for different image regions. Image classification accuracy is calculated by dividing the number of correctly classified images by the total number of test images. Computation time is rounded to one decimal place.

Region 1 (1080 × 1350 Pixels)				Region 2 (360 × 324 Pixels)				Region 3 (540 × 400 Pixels)			
Number of Blocks	Block Size	Accuracy	Time (s)	Number of Blocks	Block Size	Accuracy	Time (s)	Number of Blocks	Block Size	Accuracy	Time (s)
1 × 1 (1)	1080 × 1350	100%	0.8	1 × 1 (1)	360 × 324	100%	0.1	1 × 1 (1)	540 × 400	75%	0.1
4 × 5 (20)	270 × 270	100%	0.8	10 × 9 (90)	36 × 36	100%	0.2	27 × 20 (540)	20 × 20	100%	0.5
8 × 10 (80)	135 × 135	100%	0.9	20 × 18 (360)	18 × 18	100%	0.4	54 × 40 (2,160)	10 × 10	100%	1.5
12 × 15 (180)	90 × 90	100%	1.0	30 × 27 (810)	12 × 12	100%	0.7	108 × 80 (8,640)	5 × 5	100%	5.9
20 × 25 (500)	54 × 54	100%	1.3	40 × 36 (1,440)	9 × 9	100%	1.1	135 × 100 (13,500)	4 × 4	100%	8.9
24 × 30 (720)	45 × 45	100%	1.4	60 × 54 (3,240)	6 × 6	100%	2.3	270 × 200 (54,000)	2 × 2	100%	36.4
36 × 45 (1,620)	30 × 30	100%	2.0	90 × 81 (7,290)	4 × 4	100%	4.9	540 × 400 (216,000)	1 × 1	100%	143.1
40 × 50 (2,000)	27 × 27	100%	2.3	120 × 108 (12,960)	3 × 3	100%	8.4				
60 × 75 (4,500)	18 × 18	100%	3.8	180 × 162 (29,160)	2 × 2	100%	18.9				
72 × 90 (6,480)	15 × 15	100%	5.4	360 × 324 (116,640)	1 × 1	100%	77.5				
108 × 135 (14,580)	10 × 10	100%	10.2								
120 × 150 (18,600)	9 × 9	100%	12.8								
180 × 225 (40,500)	6 × 6	100%	27.8								
216 × 270 (58,320)	5 × 5	100%	38.8								
360 × 450 (162,000)	3 × 3	100%	110.4								
540 × 675 (364,500)	2 × 2	100%	278.3								
1080 × 1350 (1458,000)	1 × 1	100%	977.0								

Table S5. The calculated mean, maximum, and difference (maximum - mean) of the cosine similarity between VOC images (test and training datasets) of each category and their average feature vector using region 1, region 2, and region 3 images. The segmentation methods for region 1, region 2, and region 3 are 4 × 5, 40 × 36, and 27 × 20, respectively.

	Region 1 (1080 × 1350 Pixels)			Region 2 (360 × 324 Pixels)			Region 3 (540 × 400 Pixels)		
Category	Mean	Maximum	Difference ($\times 10^{-5}$)	Mean	Maximum	Difference ($\times 10^{-5}$)	Mean	Maximum	Difference ($\times 10^{-5}$)
C-17	0.999846627	0.999986109	13.9482	0.999911580	0.999967859	5.62785	0.999889408	0.999949483	6.00745
B-17	0.999691023	0.999976378	28.5355	0.999783576	0.999901696	11.8121	0.999908099	0.999945125	3.70251
A-17	0.999797279	0.999987391	19.0112	0.999855090	0.999918213	6.31233	0.999886790	0.999932741	4.59518
BC-17	0.999974023	0.999985966	1.1943	0.999924342	0.999963347	3.90049	0.999946493	0.999965085	1.85919
AC-17	0.999879841	0.999951927	7.20861	0.999842882	0.999920453	7.75705	0.999895845	0.999920341	2.4496
AB-17	0.999972724	0.999989514	1.67899	0.999937037	0.999968143	3.11056	0.999960535	0.999970369	0.983427
C-20	0.999837025	0.999929173	9.21486	0.999856168	0.999908189	5.20213	0.999859901	0.999889420	2.95189
B-20	0.999897193	0.999984994	8.78007	0.999875204	0.999935289	6.00856	0.999955348	0.999965715	1.03672
A-20	0.999942930	0.999983421	4.04908	0.999786764	0.999945222	15.8459	0.999881103	0.999942795	6.16915
BC-20	0.999661726	0.999908995	24.7269	0.999886128	0.999956428	7.03008	0.999801007	0.999896868	9.5861
AC-20	0.999969216	0.999988074	1.88588	0.999931147	0.999959116	2.79684	0.999934454	0.999950963	1.65089
AB-20	0.999979472	0.999988681	0.9209	0.999815164	0.999879182	6.40177	0.999947614	0.999959712	1.20979
C-24	0.999955404	0.999982932	2.75285	0.999922908	0.999971496	4.85884	0.999932895	0.999947431	1.45359
B-24	0.999950454	0.999985510	3.50561	0.999897823	0.999966347	6.85241	0.999479921	0.999616216	13.6295
A-24	0.999829908	0.999972105	14.2197	0.999822874	0.999960133	13.7259	0.999863046	0.999922967	5.99215
BC-24	0.999813464	0.999921821	10.8358	0.999882720	0.999936275	5.35556	0.999698831	0.999794566	9.57343
AC-24	0.999969828	0.999993885	2.40569	0.999960944	0.999985096	2.41517	0.999948019	0.999959298	1.12787
AB-24	0.999941460	0.999992703	5.12425	0.999900705	0.999964612	6.39064	0.999954715	0.999966576	1.18611
C-27	0.999925328	0.999970620	4.52928	0.999923042	0.999965452	4.24106	0.999889714	0.999920963	3.12492
B-27	0.999888502	0.999965473	7.69708	0.999808268	0.999930069	12.1801	0.999878116	0.999951101	7.29848
A-27	0.999887344	0.999936181	4.88369	0.999779142	0.999860998	8.18561	0.999865326	0.999940855	7.55287
BC-27	0.999825074	0.999987136	16.2062	0.999872129	0.999961697	8.9568	0.999913620	0.999962993	4.9373
AC-27	0.999959898	0.999991298	3.14001	0.999955700	0.999978626	2.29261	0.999919344	0.999954013	3.46687
AB-27	0.999875111	0.999978093	10.2982	0.999878617	0.999929050	5.04336	0.999946788	0.999972762	2.59733

Table S6. The calculated mean, maximum, and difference (maximum - mean) of the cosine similarity between helium plasma images (test and training datasets) of each category and their average feature vector using region 1, region 2, and region 3 images. The segmentation methods for region 1, region 2, and region 3 are 4 × 5, 40 × 36, and 27 × 20, respectively.

	Region 1 (1080 × 1350 Pixels)			Region 2 (360 × 324 Pixels)			Region 3 (540 × 400 Pixels)		
Category	Mean	Maximum	Difference ($\times 10^{-6}$)	Mean	Maximum	Difference ($\times 10^{-6}$)	Mean	Maximum	Difference ($\times 10^{-6}$)
He-17	0.999996216	0.999998878	2.66225	0.999986732	0.999988551	1.81868	0.999938206	0.999941371	3.16534
He-20	0.999998504	0.999998829	0.32491	0.999990591	0.999990850	0.259198	0.999936777	0.999939753	2.9761
He-24	0.999996558	0.999999051	2.49281	0.999990486	0.999991565	1.0795	0.999923854	0.999928694	4.84004
He-27	0.999997109	0.999998563	1.45402	0.999990560	0.999991237	0.676999	0.999919683	0.999925047	5.364

Python Code for the Cosine Similarity Method VOCs Identification using Feature Vectors Generated by Plasma Image RGB Data

Notes:

1. For the test_dir/train_dir (test_x_y/train_x_y) in the code below, x = 0 means reading and processing plasma images during VOCs injection, while x = 1 means reading and processing helium plasma images under different GC inlet helium pressures. For y (1, 2, 3), 1, 2, 3 represent region 1, 2, and 3 image, respectively.

2. Plasma images during VOCs injection and helium plasma images have the same crop size under same image region.

```
import os
import cv2
import re
import numpy as np
import pandas as pd
import time

print(u'Processing...\n')

start_time = time.time()

w_fg = 12
h_fg = 15
picflag = 24

train_dir = 'train_0_1'
test_dir = 'test_0_1/'

def readpic(fn):
    fnimg = cv2.imread(fn)
    img = cv2.resize(fnimg, (1080, 1350))
    w = img.shape[1]
    h = img.shape[0]
    w_interval = int(w / w_fg)
    h_interval = int(h / h_fg)
    alltz = []
    alltz.append([])
    alltz.append([])
    alltz.append([])
    for now_h in range(0, h, h_interval):
        for now_w in range(0, w, w_interval):
            b = img[now_h:now_h + h_interval, now_w:now_w + w_interval, 0]
            g = img[now_h:now_h + h_interval, now_w:now_w + w_interval, 1]
            r = img[now_h:now_h + h_interval, now_w:now_w + w_interval, 2]
            btz = np.mean(b)
            gtz = np.mean(g)
            rtz = np.mean(r)
            alltz[0].append(btz)
            alltz[1].append(gtz)
            alltz[2].append(rtz)
    return alltz

def get_cossimi(x, y):
    myx = np.array(x)
```

```
    myy = np.array(y)
    cos1 = np.sum(myx * myy)
    cos21 = np.sqrt(sum(myx * myx))
    cos22 = np.sqrt(sum(myy * myy))
    return cos1 / float(cos21 * cos22)

train_x = []
d = []

category_image_count = {}
pattern = re.compile(r'^(\d+)-\d+\.jpg$')

def extract_number(filename):
    match = re.match(r'^(\d+)-', filename)
    if match:
        return int(match.group(1))
    return float('inf')

for filename in sorted(os.listdir(train_dir), key=extract_number):
    if pattern.match(filename):
        category = int(filename.split('-')[0])

        if category in category_image_count:
            category_image_count[category] += 1
        else:
            category_image_count[category] = 1

for category, num_images in category_image_count.items():
    mytz = np.zeros((3, w_fg * h_fg))

    for jj in range(1, num_images + 1):
        fn = f'{train_dir}/{category}-{jj}.jpg'
        tmptz = readpic(fn)
        mytz += np.array(tmptz)

    mytz /= num_images
    train_x.append(mytz[0].tolist() + mytz[1].tolist() + mytz[2].tolist())

if picflag == len(category_image_count):
    similarity_data = {'Class': [f'Class {i + 1}' for i in range(picflag)]}
else:
    print("Adjust the value of picflag")

ordered_test_files = []

test_files = [f for f in os.listdir(test_dir) if f.endswith('.jpg') or
f.endswith('.png')]

for fn in test_files:
    full_fn = os.path.join(test_dir, fn)
    testtz = np.array(readpic(full_fn))
    simtz = testtz[0].tolist() + testtz[1].tolist() + testtz[2].tolist()
    similarity_list = []
    maxtz = 0
    nowi = 0

    for i in range(picflag):
        nowsim = get_cossimi(train_x[i], simtz)
        similarity_list.append(nowsim)
        if nowsim > maxtz:
```

```
            maxtz = nowsim
            nowi = i
    image_name = fn.rsplit('.', 1)[0]  # Remove file extension for header
    similarity_data[image_name] = similarity_list
    ordered_test_files.append(image_name)
    print(u'\n%s belongs to Class %d' % (fn, nowi + 1))

output_file = f'raw_output/{test_dir.strip("/")}.xlsx'
df = pd.DataFrame(similarity_data)
ordered_test_files = sorted(ordered_test_files, key=lambda x: int(re.match(r'^(\d+)', 
x).group(1)))
df = df[['Class'] + ordered_test_files]
df.to_excel(output_file, index=False)

print(f'\nCosine Similarity data has been exported to {output_file}')

end_time = time.time()

execution_time = end_time - start_time
print(f"\nExecution time: {execution_time:.2f} seconds")
```

References

(1) Lin, Z.; Abbott, J.; Karuso, P.; Wong, D. K. Advances in electroanalytical sensing of volatile organic compounds towards field-deployable detection. *TrAC, Trends Anal. Chem.* **2025**, *183*, 118101.
(2) Tian, Y.; Liu, Y.; Dong, K.; Zhao, B.; Tang, S.; Nie, X.; Yan, Y. Advances in rapid detection of volatile organic compounds (VOCs): From conventional techniques to surface-enhanced Raman spectroscopy. *Results Chem.* **2025**, *16*, 102329.
(3) Kanan, S.; Obeideen, K.; Moyet, M.; Abed, H.; Khan, D.; Shabnam, A.; El-Sayed, Y.; Arooj, M.; Mohamed, A. A. Recent advances on metal oxide based sensors for environmental gas pollutants detection. *Crit. Rev. Anal. Chem.* **2025**, *55* (5), 911-944.
(4) Guo, X.; Shen, J.; Liu, Z.; Guo, B. B.; Love, D.; McKinney, P. J.; Zhang, J. Experimental Evaluation of Low-Cost Metal Oxide Volatile Organic Compounds Sensors for Indoor Air Quality Monitoring. *Build. Environ.* **2026**, *291*, 114232.
(5) Liu, K.; Zhang, C. Volatile organic compounds gas sensor based on quartz crystal microbalance for fruit freshness detection: A review. *Food Chem.* **2021**, *334*, 127615.
(6) Pérez, R. L.; Ayala, C. E.; Park, J.-Y.; Choi, J.-W.; Warner, I. M. Coating-based quartz crystal microbalance detection methods of environmentally relevant volatile organic compounds. *Chemosensors* **2021**, *9* (7), 153.
(7) Cao, Y.; Fu, M.; Fan, S.; Gao, C.; Ma, Z.; Hou, D. Hydrophobic MOF/PDMS-based QCM sensors for VOCs identification and quantitative detection in high-humidity environments. *ACS Appl. Mater. Interfaces* **2024**, *16* (6), 7721-7731.
(8) Li, X.; Sun, W.; Fu, W.; Lv, H.; Zu, X.; Guo, Y.; Gibson, D.; Fu, Y.-Q. Advances in sensing mechanisms and micro/nanostructured sensing layers for surface acoustic wave-based gas sensors. *J. Mater. Chem. A* **2023**, *11* (17), 9216-9238.
(9) Zhou, H.; Ramaraj, S. G.; Sarker, M. S.; Tang, S.; Yamahara, H.; Tabata, H. Parts-per-trillion-level acetone gas detection using a suspended graphene/SiO_2 SAW breath and skin gas sensor: Simulation and experimental study. *ACS Sens.* **2025**, *10* (2), 804-813.
(10) Rezende, G. C.; Le Calvé, S.; Brandner, J. J.; Newport, D. Micro photoionization detectors. *Sens. Actuators, B* **2019**, *287*, 86-94.
(11) Cai, Y.; Che, X.; Duan, Y. From Volume to Mass: Transforming Volatile Organic Compound Detection with Photoionization Detectors and Machine Learning. *Sensors* **2025**, *25* (17), 5314.
(12) Li, Y.; Wang, Z.; Zhao, T.; Li, H.; Jiang, J.; Ye, J. Electronic nose for the detection and discrimination of volatile organic compounds: Application, challenges, and perspectives. *TrAC, Trends Anal. Chem.* **2024**, *180*, 117958.
(13) He, S.; Wen, J.; Cao, B.; Shi, G.; Zhang, M. Porous Material-Based Electronic Noses for the Sensing of Volatile Organic Compounds. *ACS Appl. Mater. Interfaces* **2025**, *17* (44), 60055-60103.
(14) Khatib, M.; Haick, H. Sensors for volatile organic compounds. *ACS Nano* **2022**, *16* (5), 7080-7115.
(15) Hernik, A.; Smets, J.; Wang, Z.; Semenova, Y.; Tan, J. C.; Ameloot, R.; Naydenova, I. Optical detection of volatile organic compounds: a review of methods and functionalized sensing materials. *Adv. Opt. Mater.* **2025**, *13* (27), e00369.
(16) Lee, Y.; Son, H.; Lee, J.; Lim, S.-H. Review on micro-gas chromatography system for analysis of multiple low-concentration volatile organic compounds: preconcentration, separation, detection,

integration, and challenges. *Micro Nano Syst. Lett.* **2024**, *12*.
(17) Lee, Y.; Lee, S.; Jang, W.; Lee, J.; Choi, Y.; Lim, S.-H. Hybrid GC platform: a micro gas chromatography system with a simple configuration for low-concentration VOC analysis. *Lab Chip* **2025**, *25* (18), 4620-4634.
(18) Thamatam, N.; Chowdhury, M.; Agah, M. Fluidic and Electrical Modular Interfacing: A modular approach to micro total analytical systems and micro gas chromatography. *Sens. Actuators, B* **2025**, *443*, 138273.
